# Comparative efficacy and safety of oral antidiabetic drugs and insulin in treating gestational diabetes mellitus: An updated PRISMA-compliant network meta-analysis

**DOI:** 10.1097/MD.0000000000007939

**Published:** 2017-09-22

**Authors:** Hui-ling Liang, Shu-juan Ma, Yan-ni Xiao, Hong-zhuan Tan

**Affiliations:** Department of Epidemiology and Statistics, Xiangya School of Public Health, Central South University, Changsha, Hunan, China.

**Keywords:** gestational diabetes mellitus, glyburide, insulin, metformin, network meta-analysis, treatment

## Abstract

**Background:**

The safety and efficacy of different drugs in treatment of gestational diabetes mellitus (GDM) patients who could not maintain normal glucose level only through diet and exercise remains to be debated. We performed this network meta-analysis (NAM) to compare and rank different antidiabetic drugs in glucose level control and pregnancy outcomes in GDM patients.

**Methods:**

We searched PubMed, Cochrane Library, Web of Science, and Embase up to December 31, 2016. Randomized controlled trials (RCTs) related to different drugs in the treatment of GDM patients were enrolled. We extracted the relevant information and assessed the risk of bias with the Cochrane risk of bias tool. We did pair-wise meta-analyses using the fixed-effects model or random-effects model and then adopted random-effects NAM combining both direct and indirect evidence within a Bayesian framework, to calculate the odds ratio (OR) or standardized mean difference (SMD) and to draw a surface under the cumulative ranking curve of the neonatal and maternal outcomes of different treatments in GDM patients.

**Results:**

Thirty-two randomized controlled trials (RCTs) were included in this NAM, including 6 kinds of treatments (metformin, metformin plus insulin, insulin, glyburide, acarbose, and placebo). The results of the NAM showed that regarding the incidence of macrosomia and LGA, metformin had lower incidence than glyburide (OR, 0.5411 and 0.4177). In terms of the incidence of admission to the NICU, insulin had higher incidence compared with glyburide (OR, 1.844). As for the incidence of neonatal hypoglycemia, metformin had lower incidence than insulin and glyburide (OR, 0.6331 and 0.3898), and insulin was lower than glyburide (OR, 0.6236). For mean birth weight, metformin plus insulin was lower than insulin (SMD, -0.5806), glyburide (SMD, -0.7388), and placebo (SMD, -0.6649). Besides, metformin was observed to have lower birth weight than glyburide (SMD, 0.2591). As for weight gain, metformin and metformin plus insulin were lower than insulin (SMD, -0.9166, -1.53). Ranking results showed that glyburide might be the optimum treatment regarding average glucose control, and metformin is the fastest in glucose control for GDM patients; glyburide have the highest incidence of macrosomia, preeclampsia, hyperbilirubinemia, neonatal hypoglycemia, shortest gestational age at delivery, and lowest mean birth weight; metformin (plus insulin when required) have the lowest incidence of macrosomia, PIH, LGA, RDS, low gestational age at delivery, and low birth weight. Besides, insulin had the highest incidence of NICU admission, acarbose had the lowest risk of neonatal hypoglycemia.

**Conclusion:**

Our study concluded that metformin is fastest in glucose control, with a more favorable pregnancy outcomes—would be a better option, but its rate of glucose control is the lowest.However, glyburide is the optimumtreatment regarding the rate of glucose control, but withmore adverse outcomes. This NAMbased on 32 RCTs will strongly help to guide further development of management for GDM patients, clinicians should carefully balance the risk–benefit profile of different treatments according to various situations.

## Abstract 2

Supplemental Digital Content is available in the text

## 1 Introduction

Gestational diabetes mellitus (GDM) is a major global public health issue, with prevalence increasing in recent years due to the epidemic of obesity and type 2 diabetes.^[1,2]^

GDM is defined as a condition in which a woman without diabetes develops the glucose intolerance resulting in hyperglycemia of variable degree during pregnancy.^[3]^ Risk factors of developing GDM include being overweight, polycystic ovary syndrome, maternal age, and a family history with type 2 diabetes. GDM generally exhibit no symptoms, but it increases the risk of preeclampsia, depression, and the incidence of cesarean section. Moreover, children born to mothers with badly treated GDM are at higher risk of LGA, hypoglycemia, jaundice or at increased risk of being overweight and developing type 2 diabetes.^[4]^ So the management of GDM is primarily aimed at glycemic control to reduce the incidence of adverse pregnancy outcomes.^[5]^

Most women are able to control their blood sugar with proper diet or plus exercise, if not, insulin treatment is considered as the gold standard for GDM.^[6,7]^ However, several disadvantages of insulin treatment are recognized such as frequent injections, increased risk of hypoglycemia, and higher cost,^[8]^ which could reduce patient's compliance. Furthermore, the dose of insulin needs to be individualized according to the women's body mass index (BMI), glucose control levels, and lifestyle.^[9]^ By contrast, oral agents (metformin and glyburide) present the advantages of easier management and lower cost, so that they become an attractive alternative to insulin with better acceptance,^[10]^ which enhance adherence to the treatment.^[11]^ Metformin is a biguanide that achieves euglycemia primarily by suppressing hepatic gluconeogenesis and enhancing peripheral glucose uptake.^[12]^ Glyburide acts by binding to and inhibiting the ATP-sensitive potassium channels (KATP) in pancreatic beta cells, and leads to an increase in intracellular calcium in the beta cell and subsequent stimulation of insulin secretion.^[13]^

Several previous studies have compared efficacy and safety of oral antidiabetic drugs (OADs) and insulin in treating GDM, with somewhat inconsistent results. A recent meta-analysis^[14]^ including 11 RCTs found metformin was comparable with insulin in glycemic control, and could significantly reduce several adverse pregnancy outcomes. Another study^[15]^ suggested that glyburide is as effective as insulin, but the risk of macrosomia, neonatal hypoglycemia, and fetal birth weight were higher. However, there is 1 RCT^[16]^ concluded that metformin was equivalent to glyburide both for women and newborns. Moreover, another review^[17]^ mentioned that glyburide is more effective in lowering blood sugar in women with GDM, and with a lower treatment failure rate than metformin. Therefore, there is still debate about which would be the most favorable hypoglycemic drugs in GDM patients.

In recent years, several previous traditional meta-analyses (TMAs) have been performed to compare the efficacy and safety of OADs with insulin. Nevertheless, the results were inconsistent due to the lack of evidence from head-to-head RCTs. However, network meta-analysis (NMA), also known as mixed treatment comparisons (MTC), allows to compare more than 2 treatments (e.g., treatments A, B, C), by including both direct and indirect comparisons, and thereby making it possible to rank all the treatments, and to pool all the available evidence.^[18,19]^ In 2014, one NMA^[20]^ including 18 RCTs revealed that both metformin and glyburide are suitable for use in the management of GDM, but glyburide was associated with more adverse pregnancy outcomes. However, there are increasing number of new clinical trials conducted to evaluate the relative efficacy and safety of OADs in GDM, we therefore performed an updated NMA to provide a more comprehensive assessment for available treatments by incorporating additional trials published since the last review. The NMA presented here aimed to provide more powerful evidence about the efficacy and safety of different treatments in GDM.

## 2 Materials and methods

### 2.1 Ethnic statement

The meta-analysis was based on previous published studies, thus no ethical approval and patient consent are required.

### 2.2 Search strategy and selection criteria

We searched the databases including Medline, PubMed, Embase, Cochrane Library (last search was updated on December 31, 2016). The terms used to search were “Gestational Diabetes” or “GDM” and “oral hypoglycemic agents,” “oral antidiabetic drugs,” “glibenclamide,” “metformin,” “glyburide,” or “acarbose,” in combination with RCT. Finally, we searched for additional eligible trials in reference lists of retrieved publications and relevant meta-analyses.

Studies were included if they met the following criteria: subjects were women with gestational diabetes requiring drug treatment; randomized control trials (RCTs) of comparing efficacy and safety parameters of different OADs or OADs versus insulin for GDM; studies offering information at least 1 maternal or fetal outcome; maternal outcomes were glycohemoglobin (HbA1c), fasting blood glucose (FBG), 2-hour postprandial glucose (2HPG), pregnancy-induced hypertension (PIH), weight gain and preeclampsia; neonatal outcomes were hypoglycemia, mean birth weight, macrosomia, large for gestational age (LGA), preterm birth, neonatal intensive care unit (NICU), hyperbilirubinemia, respiratory distress syndrome (RDS) and gestational age at delivery. The exclusion criteria were as follows: Reviews, letters, and comments were excluded; studies published with insufficient information; duplicate studies were excluded, in the case that significant overlap with multiple publications by the same group; studies involving pregnant women with preexisting diabetes were excluded. No language restrictions were set.

### 2.3 Data collection and quality assessment

Two investigators independently reviewed trials for eligibility and extracted relevant information from included trials with a standard protocol, and assessed the risk of bias with the Cochrane risk of bias tool.^[21]^ We extracted study characteristics (author name, publication year, country, BMI of study subjects, sample size), intervention, outcomes (maternal and neonatal outcomes), and risk of bias. Any disagreements between reviewers were resolved by discussion.

### 2.4 Outcomes of interest

Outcomes of interest were divided into 2 categories: neonatal outcomes and maternal outcomes. Neonatal outcomes included macrosomia, LGA births, hypoglycemia, mean birth weight, neonatal intensive care unit (NICU), hyperbilirubinemia, RDS, gestational age at delivery. Maternal outcomes included glycohemoglobin (HbA1c), FBG, 2HBG, PIH, weight gain, and preeclampsia. The endpoint definitions as applied in each trial were incorporated.

### 2.5 Statistical analysis

#### 2.5.1 Pairwise meta-analysis

We conducted pairwise meta-analyses with a fixed effects model or random effects model. The standardized mean difference (SMD) was calculated as the effect size for continuous variables and the odds ratio (OR) was calculated for dichotomous variables, both with 95% CI. The *I*^2^ statistic and *P*-value was used to quantify heterogeneity in each pairwise comparison. *I*^2^ > 50% or *P* < .01 indicated the existence of heterogeneity across the studies.^[21]^ The Egger test was used to detect publication bias. All statistical analysis was conducted using STATA version 12.0 (Stata Corp, College Station, TX).

#### 2.5.2 Network meta-analysis (NMA)

The Bayesian NMA is a generalization of pair-wise meta-analysis, which was performed within Bayesian inference with the use of Gibbs sampling methods that allow combined direct and indirect comparisons. An advantage of this approach is that it is straightforward to extend to shared parameter models where different RCTs outcomes in different formats but from a common underlying model.^[18]^ Then, a random-effects model was selected to allow for heterogeneity among trials on the assumption that different treatment effects originated from a normal distribution. Bayesian inference with WinBUGS software (version 1.4.3, MRC Biostatistics Unit, Cambridge, UK)^[21]^ uses Markov Chain Monte Carlo (MCMC) simulation to calculate the posterior distributions within the framework of the chosen model and likelihood function and on the basis of some prior assumptions.^[22,23]^

Further analysis performed using R version 3.3.1 (The R Foundation for Statistical Computing) and STATA 12.0 software (Stata Corp).^[24]^ The results of NMA with effect sizes (SMD or OR) and their credible intervals (CI) were obtained by the MCMC method. See Appendix for details about the WinBUGS codes used. Three Markov chains ran simultaneously with different initial values chosen arbitrarily, with 40,000 iterations, and the first 10,000 simulations were discarded due to the burn-in period.^[25,26]^ A network plot was drawn with the nodes representing interventions, the node size representing sample sizes, and the line thicknesses indicating the available direct comparisons between pairs of interventions.

We did the inconsistency analysis with RoR (the ratio of 2 ORs) values in every closed loop and drawn inconsistency plot to assess inconsistency between direct and indirect sources of evidence. RoR values close to 1 mean that the 2 sources are in agreement.^[27]^

The surface under the cumulative ranking curve (SUCRA) is used to provide a hierarchy of the treatments. The SUCRA value was presented as the percentage of the area under the curve, the larger the SUCRA value, the better the treatment or the lower the incidence of adverse effects. The presence of small-study effects in a meta-analysis is assessed by comparison-adjusted funnel plot.^[28]^

## 3 Results

### 3.1 Characteristics of the included studies

**Figure 1:**
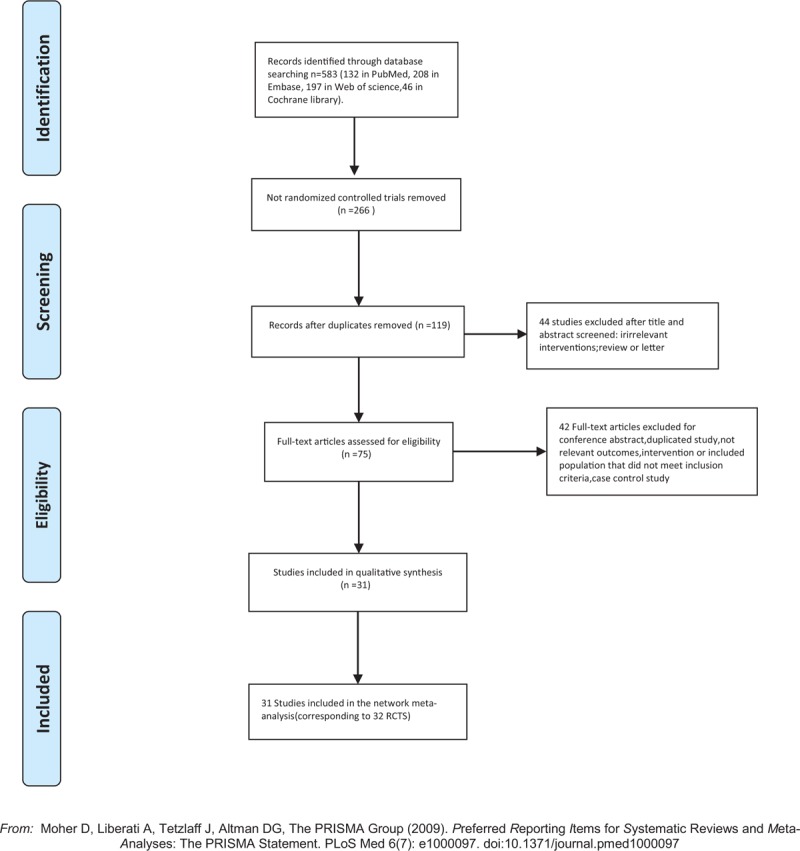
Flow diagram of study selection.

**Figure 2:**
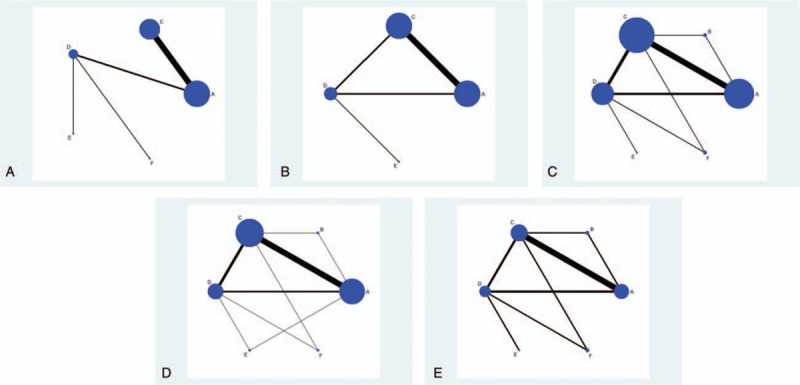
(A–E) The network plot of eligible comparisons for different treatments.

Figure 1 shows the study selection process of included trials. A total of 583 studies were initially identified by literature research, among which 464 studies were excluded for not RCTs or duplicated studies. Then after screening titles, abstracts, and full text, 86 studies were discarded because of irrelevant interventions, review or letter, duplicated study, not relevant outcomes, intervention or included population that did not meet inclusion criteria, case control study. Eventually, we enrolled 31 studies^[16,29–58]^ (corresponding to 32 RCTs) in the NMA. The characteristics of the included studies are presented in Table 1. Of all these 32 RCTs, 30 were 2-arm trials and 2 were 3-arm trials, with a total of 4723 GDM patients were enrolled. Among them, 10 and 13 RCTs reported patients with or without obesity (defined as BMI ≥ 30), respectively, the rest of 8 RCTs did not mention clearly. Subjects involved in this meta-analysis were treated with metformin (A), metformin plus insulin (B), insulin (C), glyburide (D), placebo (E), and acarbose (F). Figure 2A–E and Appendix Fig. 1 show the network plot of eligible comparisons for different treatments, and contribution plot are shown in Appendix Fig. 2.

**Table 1:**
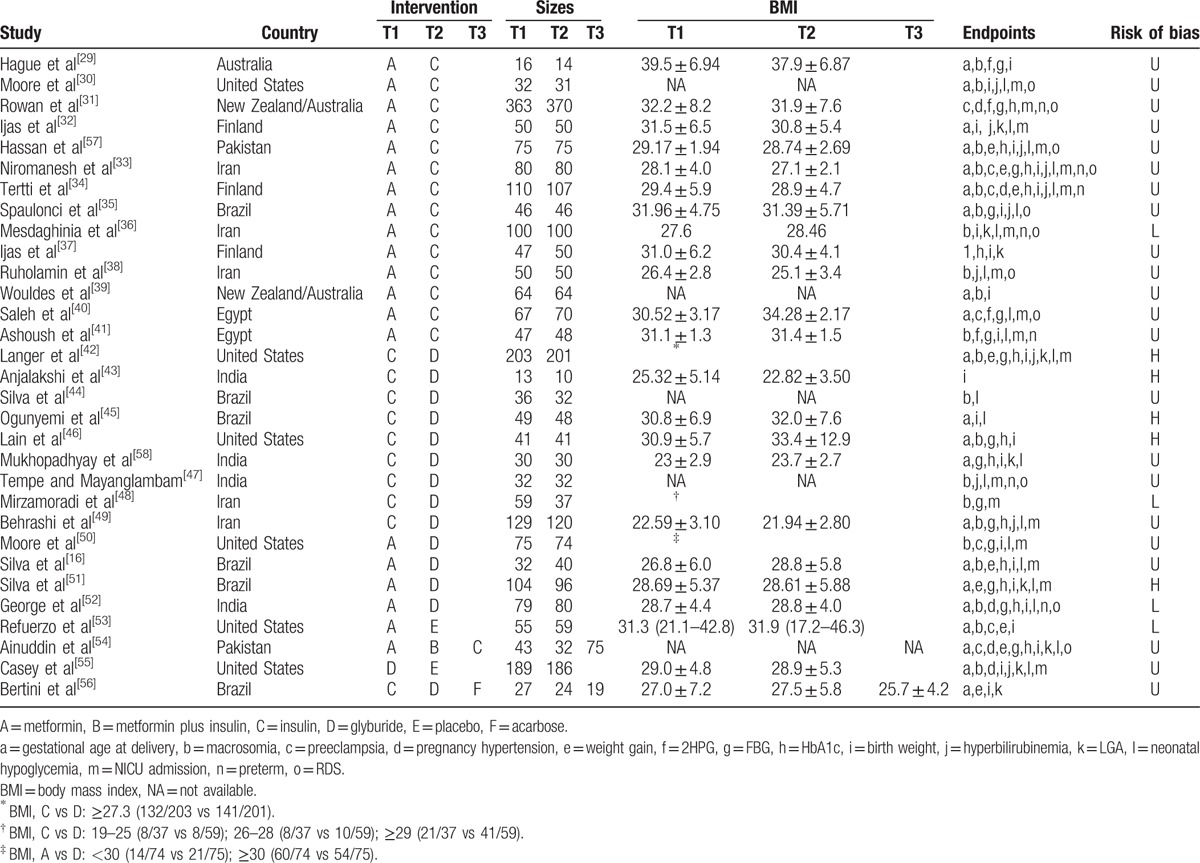
Main characteristics of the randomized trials included in the network meta-analysis.

### 3.2 Results from pairwise meta-analysis and network meta-analysis

The results of the Pairwise meta-analysis and NMA are presented as a league table in Table 2 and Appendix Tables 1 and 2.

**Table 2:**
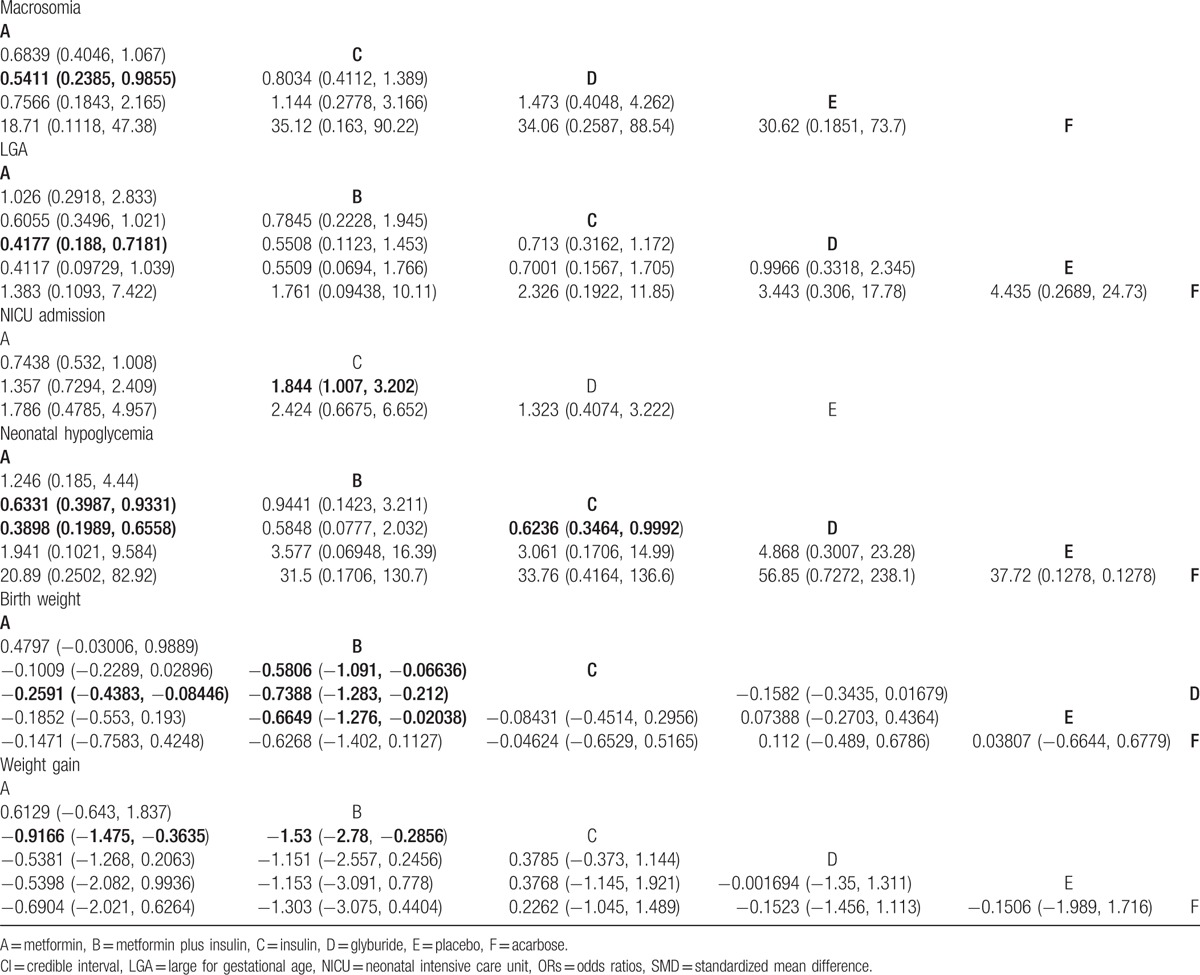
ORs or SMD and 95% CI of 6 treatments according to the network meta-analysis.

### 3.3 Macrosomia

Twenty-five studies involving 3412 GDM patients reported the macrosomia. In the pairwise meta-analysis, insulin showed no statistical significance compared with glyburide (OR, 0.788; 95% CI, 0.510–1.219); metformin was significantly lower compared with insulin (OR, 0.729; 95% CI, 0.545–0.974), but had no significant difference compared with glyburide (OR, 0.587; 95% CI, 0.239–1.442).

The NMA revealed that metformin was significantly lower compared with glyburide (OR, 0.5411; 95% CI, 0.2385–0.9855), but there were no significance between metformin and insulin or insulin and glyburide.

### 3.4 LGA

Fifteen studies involving 1813 GDM patients reported the incidence of LGA. In the pairwise meta-analysis, metformin was significantly lower than insulin (OR, 0.647; 95% CI, 0.438–0.956), and glyburide (OR, 0.431; 95% CI, 0.229–0.814), but had no significant difference between insulin and glyburide (OR, 0.838; 95% CI, 0.542–1.295).

In the NMA, metformin was observed to have lower incidence of LGA than glyburide (OR, 0.4177; 95% CI, 0.188–0.7181). No other significant results were observed about the incidence of LGA.

### 3.5 Preterm

Nine studies involving 1879 GDM patients were involved in the analysis of incidence of preterm. In the pairwise meta-analysis, metformin showed a significant increase compared with glyburide (OR, 2.887; 95% CI, 1.087–7.666), but showed no significance compared with insulin (OR, 1.332; 95% CI, 0.939–1.890).

In the NMA, we did not find any significant results about the incidence of preterm.

### 3.6 Admission to the NICU

Eighteen studies involving 3635 GDM patients focused on the incidence of admission to the NICU. In the pairwise meta-analysis, we only observed that metformin has a lower incidence of admission to the NICU than insulin (OR, 0.772; 95% CI, 0.644–0.927).

In the NMA, glyburide had significant lower incidence of admission to the NICU compared with insulin (OR, 0.542; 95% CI, 0.312–0.993), no other significant results were found.

### 3.7 Neonatal hypoglycemia

Twenty-six studies involving 3360 GDM patients reported the incidence of neonatal hypoglycemia. In the pairwise meta-analysis, metformin had lower incidence of neonatal hypoglycemia than insulin (OR, 0.636; 95% CI, 0.486–0.832), and insulin was lower than glyburide (OR, 0.647; 95% CI, 0.423–0.991).

In the NMA, metformin was significantly lower compared with insulin (OR, 0.6331; 95% CI, 0.3987–0.9331), and glyburide (OR, 0.3898; 95% CI, 0.1989–0.6558). Besides, insulin was significantly lower than glyburide (OR, 0.6236; 95% CI, 0.3464–0.9992). No other significant results were found.

### 3.8 Birth weight

Thirty studies involving 4060 GDM patients reported the mean birth weight. In the pairwise meta-analysis, metformin was significantly lower than insulin (SMD, −0.111; 95% CI, −0.194 to −0.028), and glyburide (SMD, −0.235; 95% CI, −0.399 to −0.071); insulin was significantly lower compared with glyburide (SMD, −0.180; 95% CI, −0.327 to −0.033).

In the NMA, we observed that metformin plus insulin has lower birth weight than insulin, glyburide and placebo (SMD, −0.5806; 95% CI, −1.091 to −0.06636; SMD, −0.7388; 95% CI, −1.283 to −0.212; and SMD, −0.6649; 95% CI, −1.276 to −0.02038, respectively). Besides, metformin was observed to have significantly lower birth weight than glyburide (SMD, −0.2591; 95% CI, −0.4383 to −0.08446).

### 3.9 2-hour postprandial glucose (2HPG)

Six studies involving 1345 GDM patients focused on the 2HPG. In the pairwise meta-analysis, metformin showed lower 2HPG than insulin (SMD, −0.285; 95% CI, −0.417 to −0.154), and insulin was lower than glyburide (SMD, −0.302; 95% CI, −0.493 to −0.111). In the NMA, there were no significant results.

### 3.10 Fasting blood glucose (FBG)

Seventeen studies involving 2769 GDM patients reported the FBG. In the pairwise meta-analysis, only metformin showed higher FBG than glyburide (SMD, 0.192; 95% CI, 0.018–0.366). No other significant results were observed. In the NMA, however, we did not get significant results between groups.

### 3.11 Glycohemoglobin (HbA1c)

Seventeen studies involving 2887 GDM patients reported the HbA1c. However, we did not obtain significant results from pairwise meta-analysis or NMA.

### 3.12 Gestational age at delivery

Twenty-seven studies involving 4146 GDM patients focused on the gestational age at delivery. In the pairwise meta-analysis, metformin and metformin plus insulin were significantly lower than insulin (SMD, −0.126; 95% CI, −0.212 to −0.040; SMD, −0.284; 95% CI, −0.521 to −0.048, respectively), and insulin was significantly lower than glyburide (SMD, −0.180; 95% CI, −0.303 to −0.057). In the NMA, no significant difference was identified between groups.

### 3.13 Weight gain

Fourteen studies involving 1893 GDM patients were enrolled in the analysis of weight gain. In the pairwise meta-analysis, metformin was significantly lower compared with insulin (SMD, −0.774; 95% CI, −0.928 to −0.620), and glyburide (SMD, −0.321; 95% CI, −0.560 to −0.081).

In the NMA, metformin and metformin plus insulin were observed to have significantly lower weight gain than insulin (SMD, −0.9166; 95% CI, −1.475 to −0.3635; SMD, −1.53; 95% CI, −2.78 to −0.2856, respectively). No other significant differences were observed about the weight gain.

### 3.14 Other outcomes

Thirteen studies involving 2008 GDM patients were enrolled in the analysis of the incidence of RDS, 10 studies involving 1906 patients focused on the incidence of hyperbilirubinemia, and 17 studies involving 2887 patients reported HbA1c. Besides, 7 studies involving 1634 patients were included in the analysis of PIH, 11 studies involving 1754 patients regarding the incidence of preeclampsia. However, both pairwise meta-analysis and NMA results show no significant differences between groups among these outcomes.

### 3.15 Relative ranking of 6 kinds of treatments in GDM patients

We compared the relative rank probabilities of different treatments based on SUCRA values (Table 3) and cumulative probability plots (Appendix Fig. 3A–O). The larger the SUCRA value, the better the rank of the treatment or the lower the incidence of adverse effects. According to the result, metformin ranked the best with the lowest incidence of macrosomia, 2HPG, LGA, and RDS; metformin plus insulin ranked the best regarding the risk of PIH, gestational age at delivery, weight gain, mean birth weight, and FBG; glyburide ranked the worst regarding the risk of macrosomia, preeclampsia, hyperbilirubinemia, neonatal hypoglycemia, and gestational age at delivery and mean birth weight, but ranked the best regarding the risk of NICU admission and HbA1C. Besides, insulin ranked the worst regarding the incidence of NICU admission. Acarbose ranked the best regarding the risk of neonatal hypoglycemia.

**Table 3:**
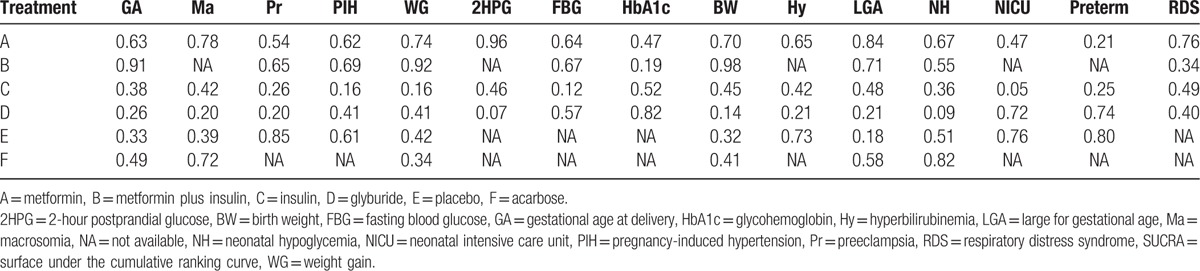
SUCRA values of 6 treatments under different pregnancy outcomes.

### 3.16 The efficacy of OADs between GDM patients with and without obesity

Ten studies involving 1577 obese GDM patients were enrolled in the analysis of the efficacy of OADs. As for 2HPG, metformin ranked the best, followed by insulin and glyburide. For FBG, metformin also ranked the best, followed by glyburide and insulin. However, regarding HbA1c, glyburide ranked the best, followed by metformin and insulin.

Thirteen studies involving 2035 nonobese GDM patients were enrolled focusing on the efficacy of OADs. As for FBG, insulin ranked the best, followed by glyburide, metformin ranked the worst. For HbA1c, glyburide ranked the best, followed by insulin, metformin ranked the worst. Detailed results are shown in Appendix Tables 3 and 4.

### 3.17 Publication bias

**Figure 3:**
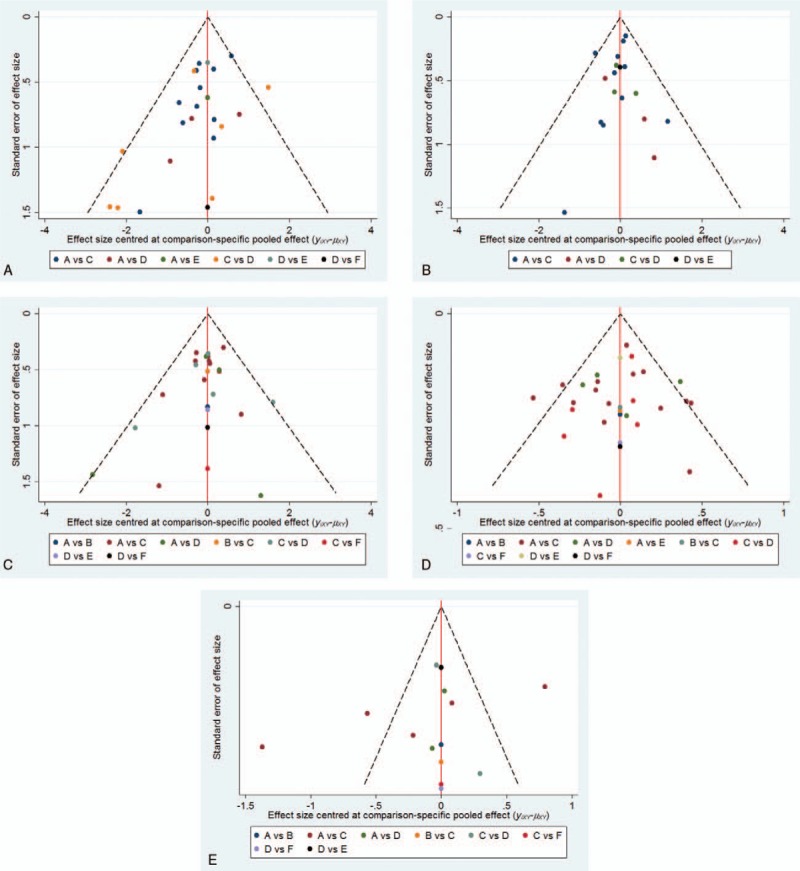
(A–E) The comparison-adjusted funnel plot from network meta-analysis.

As suggested by the Egger test (Appendix Table 1) and comparison-adjusted funnel plot for each outcome from NMA (Fig. 3A–E and Appendix Fig. 4), there was no significant publication bias among various studies.

### 3.18 Comparisons between pairwise meta-analysis and network meta-analysis

The results of pairwise meta-analysis and NMA are shown in Table 2 and Appendix Tables 1 and 2. Although the pooled estimates for the outcome showed minor differences, the confidence intervals from pairwise meta-analysis and NMA are generally consistent in majority comparisons. Tests of inconsistency showed that there was no significant inconsistency between direct and indirect comparisons (Appendix Fig. 5A–K).

## 4 Discussion

The purpose of this NMA was to evaluate the efficacy and safety of all commonly used pharmaceutical treatment for GDM, compare with each other, and rank them. In most of our included studies, we observed that the baseline BMI were slightly higher in metformin group, however, there was no statistical significance in all but one of the baseline BMI. The mean BMI of the GDM patients in Moore et al show that a significant number of them are obese in metformin group. Our findings show that glyburide might be the optimum treatment regarding average glucose control, and metformin is the fastest in glucose control for GDM patients. Then, we further explored the efficacy of OADs between GDM patients with and without obesity, and found that in obese GDM patients, metformin is generally superior to glyburide and insulin, but for nonobese GDM patients, glyburide is better than insulin and metformin, which is supported by a previous systematic review.^[59]^ Moreover, glyburide ranked the worst with the highest incidence of macrosomia, preeclampsia, hyperbilirubinemia, neonatal hypoglycemia, preterm birth, and low birth weight; metformin (plus insulin when required) has the lowest risk of macrosomia, pregnancy hypertension, LGA, RDS, preterm birth, and low birth weight. Besides, insulin had the highest incidence of NICU admission, acarbose had the lowest risk of neonatal hypoglycemia. These findings are an important addition to our knowledge about which drugs are most optimal in treatment of GDM patients.

Our findings confirm and extend previous focused studies, but go beyond them, because the network technique makes us can synthesize the data from trials with more than 2 interventions, incorporate both direct and indirect evidence, increases the accuracy in the estimates, and produces a relative rank for all kinds of treatments.^[28,60]^ All previous meta-analyses^[15,20,61,62]^ drawn the conclusion that OADs and insulin are comparable in glucose control simply from pairwise meta-analysis. In our paper, we firstly adopted network technique to combine and rank all kinds of treatments for GDM patients from different variables. For 2HPG and FBG, metformin (plus insulin when required) ranked the best indicating it reached glucose targets sooner; but for HbA1c, glyburide ranked the best, followed by insulin, metformin ranked the worst; However, FBG and 2-hour postprandial blood glucose are susceptible to eating, glucose metabolism and other related factors, merely reflecting the level of blood sugar in a specific time. HbA1c can be stable and reliable to reflect the average blood glucose level within 120 days, which has become the gold standard for diabetes monitoring. Thus, our finding suggests glyburide might be the optimum treatment regarding average glucose control, and metformin is the fastest in glucose control for GDM patients. However, every treatment may have some extent failure rate in glucose control,^[30]^ and the failure of treatment was related to the severity of GDM. Thus, clinicians should also inform patients the risk of failure when choose to utilize OADs.

In terms of glyburide, it ranked the worst with highest risk of macrosomia, preeclampsia, hyperbilirubinemia, neonatal hypoglycemia, and higher gestational age at delivery and mean birth weight. Previous reviews^[20,63]^ also found glyburide had increased incidence of macrosomia than metformin.

Moreover, metformin (plus insulin when required) ranked the best with the lowest incidence of macrosomia, PIH, LGA, RDS. But in terms of preterm, metformin ranked the worst with the highest risk of preterm birth. Furthermore, as for NICU admission, previous meta-analyses^[14,64,65]^ showed that metformin presented significantly lower incidence of NICU compared with insulin, which is in line with our pairwise meta-analysis (RR, 0.772; 95% CI, 0.644–0.927), did not provide more detailed results about glyburide. However, in our study, we found insulin has the highest risk of NICU admission, followed by metformin, glyburide ranked the best in reducing the risk of NICU admission. Besides, acarbose ranked the best in reducing the risk of neonatal hypoglycemia, followed by metformin (plus insulin when required), insulin, and glyburide.

Jiang et al^[20]^ reported a NMA result about GDM pharmaceutical treatment that also integrate direct and indirect evidence, which were not completely coincident with our results. Their analysis included fewer interventions than did in our analysis; have not included the intervention of metformin plus insulin and the outcomes of RDS and hyperbilirubinemia; and have not presented the contribution plot and cumulative probability plot. The most important is that we added new RCTs and ranked all the treatments in various outcomes, our results were more detailed and maybe more reliable.

To our knowledge, this is the largest and most comprehensive synthesis of data to date for available pharmacological treatments for GDM patients. The NMA synthesizes direct and indirect evidence that allowed comparison of all available treatments for GDM and ranking them in a single analysis, rather than separate and disconnected meta-analyses for individual pairs of treatments, which increases the precision in the estimates.^[66]^ Thus, results from NMAs are more likely to be helpful to clinicians when making choices among multiple alternatives.

Several limitations are worth noting. First, despite the sample size of the present study is largest up to date, we can only analyze 12 outcomes reported in the original RCTs and do not consider every possible relevant outcome because of too few studies were included and a small number of events. Second, indirect evidence is susceptible to confounding,^[67]^ and thus should be regarded with caution since it does not always consistent with the corresponding direct estimates.^[68,69]^ However, our analysis yielded low heterogeneity and little evidence for inconsistency. Third, we cannot explore treatment outcomes in different ethnicity without access to individual patient data. Fourth, to reduce heterogeneity, we enrolled only trials comparing among OADs or insulin, excluding trials comparing other treatment strategy.

In conclusion, metformin have more favorable pregnancy outcomes and the fastest rate of glucose control, especially in obese GDM patients, but with lowest rate of average glucose control; glyburide have the highest rate of average glucose control, particularly in nonobese GDM patients, but with more adverse outcomes. Clinicians should carefully balance the risk and benefit of different treatments according to various situations in selecting different GDM treatment strategy.

## Acknowledgment

The authors thank all the anonymous reviewers and editors for their suggestions, which will be helpful for us to improve our paper.

## Supplementary Material

Supplemental Digital Content
