# Supplementary material for: Comparative efficacy and safety of oral antidiabetic drugs and insulin in treating gestational diabetes mellitus: An updated PRISMA-compliant network meta-analysis
Source: Medicine (Baltimore). 2017 Sep 22;96(38):e7939. doi: 10.1097/MD.0000000000007939 (PMC5617694; doi:10.1097/MD.0000000000007939)
Supplement: Supplemental Digital Content [file medi-96-e7939-s001.doc]

Appendix Figure 1:

Network plot of the comparisons for the Bayesian network meta-analysis.


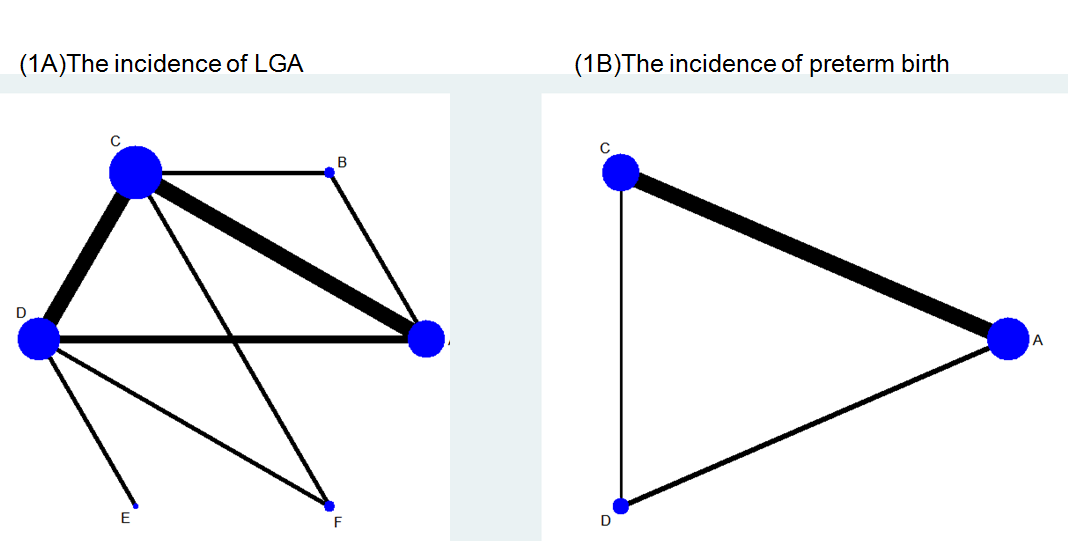


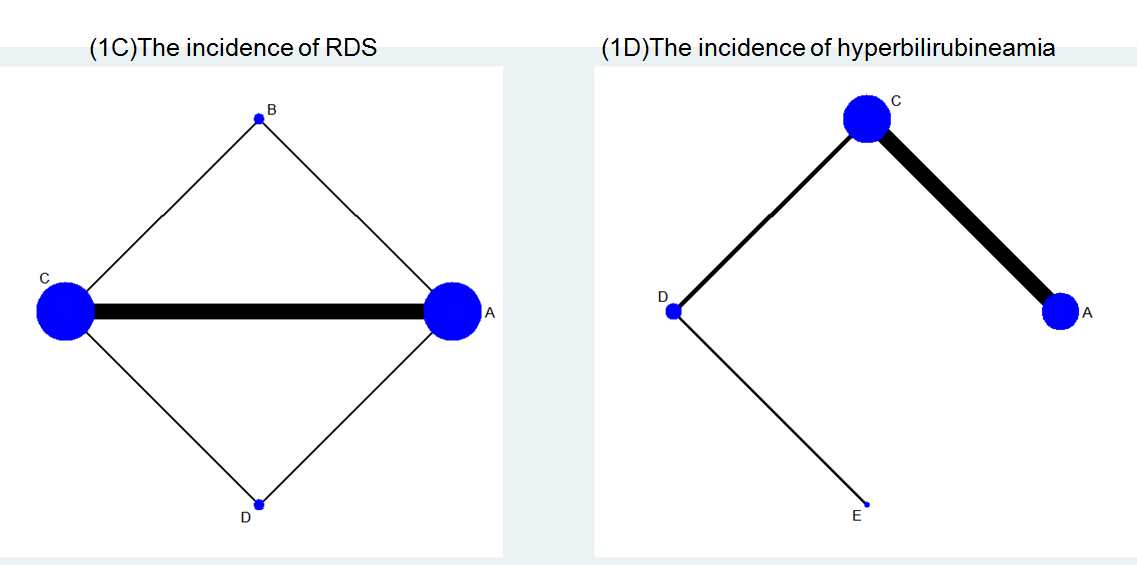


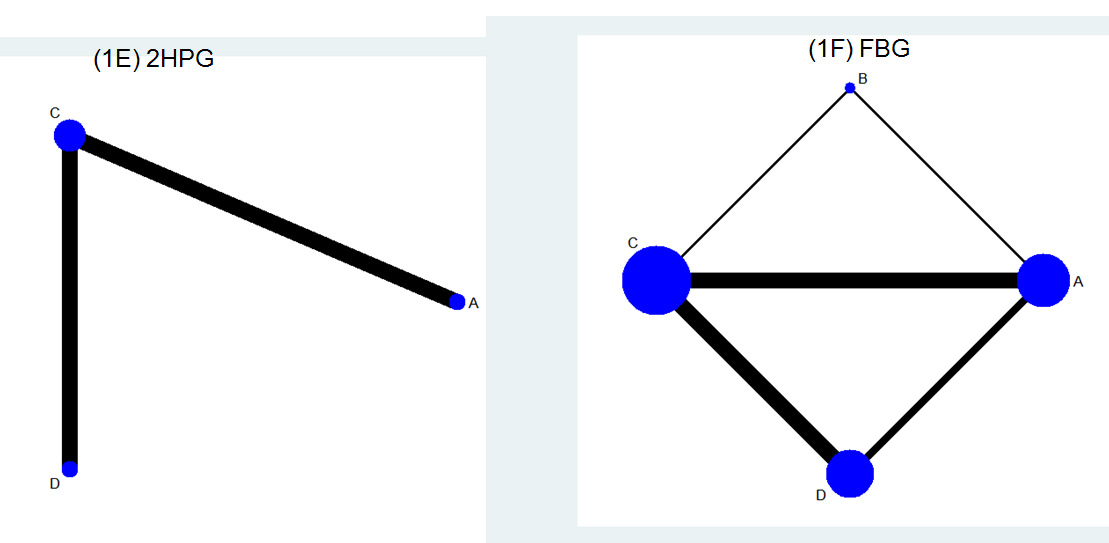


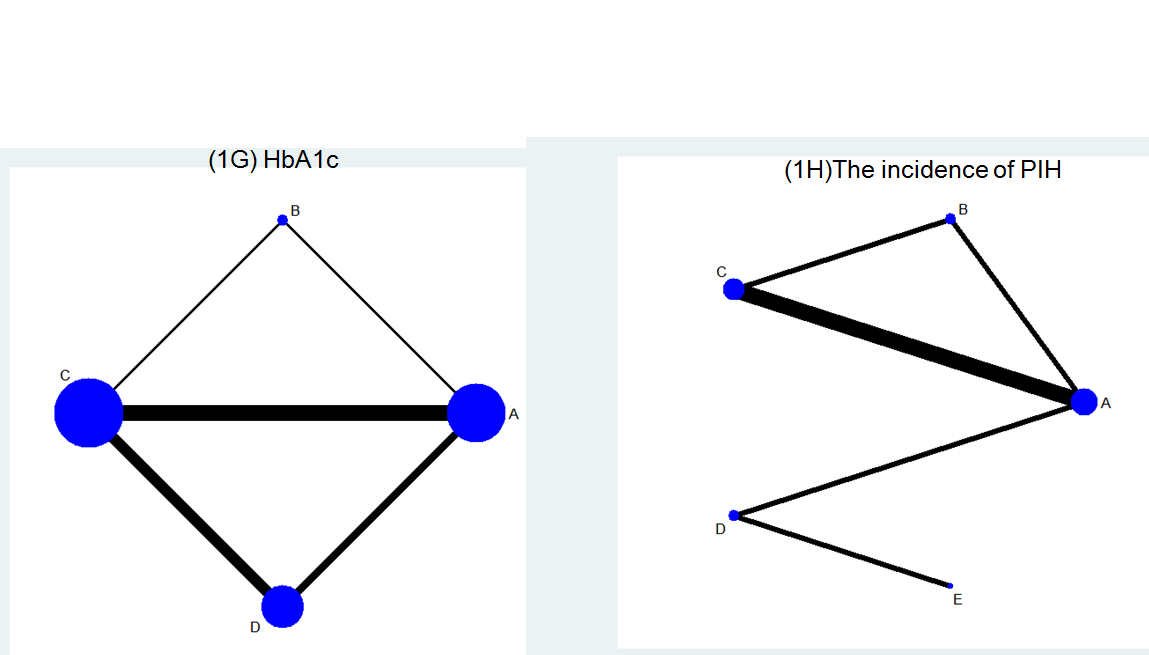


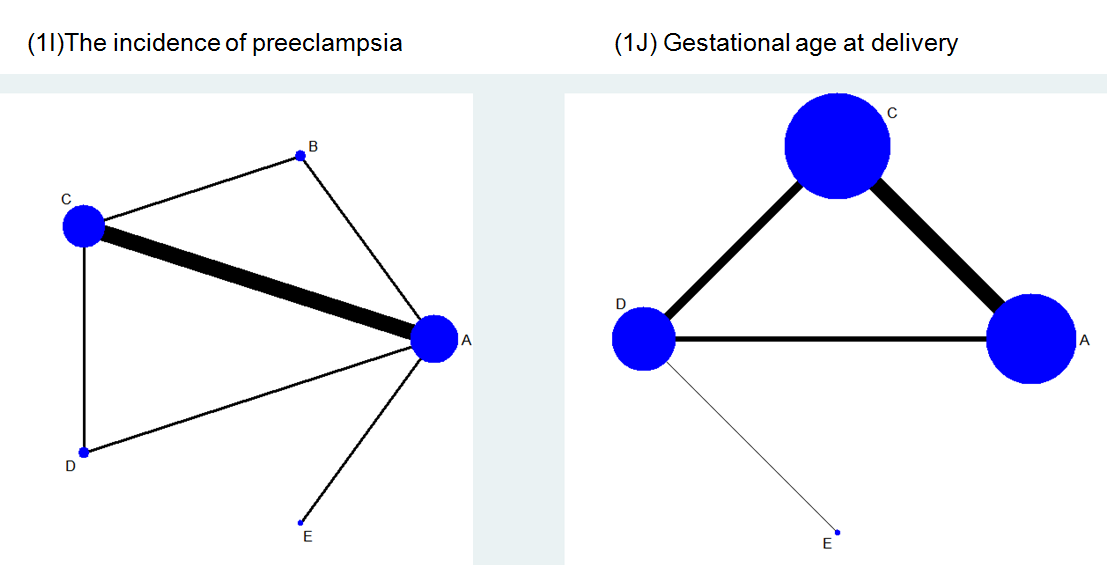


Appendix Figure 2：Contribution plot of the comparisons for the Bayesian network meta-analysis.


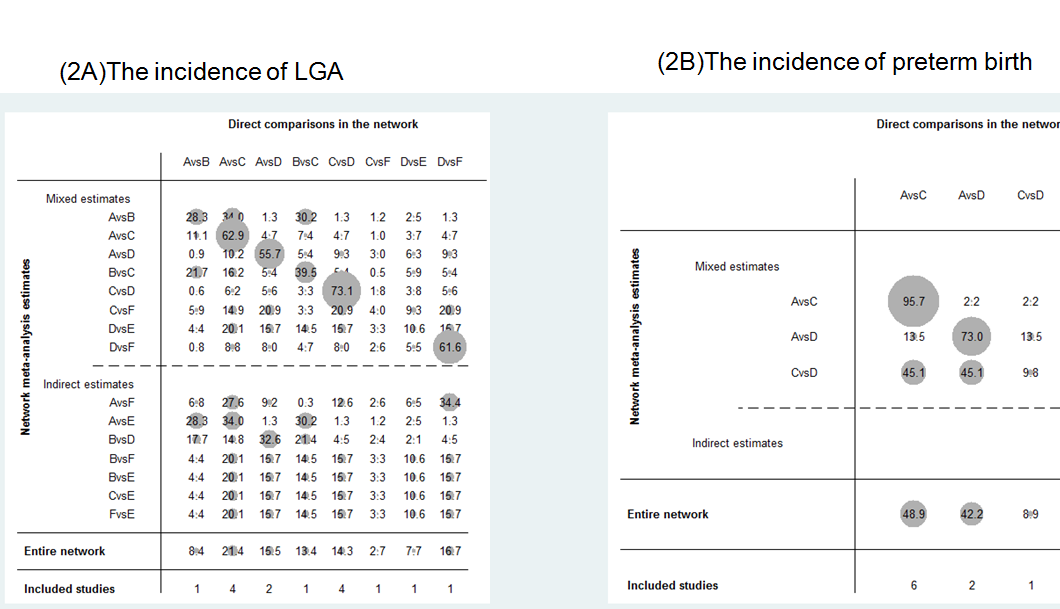


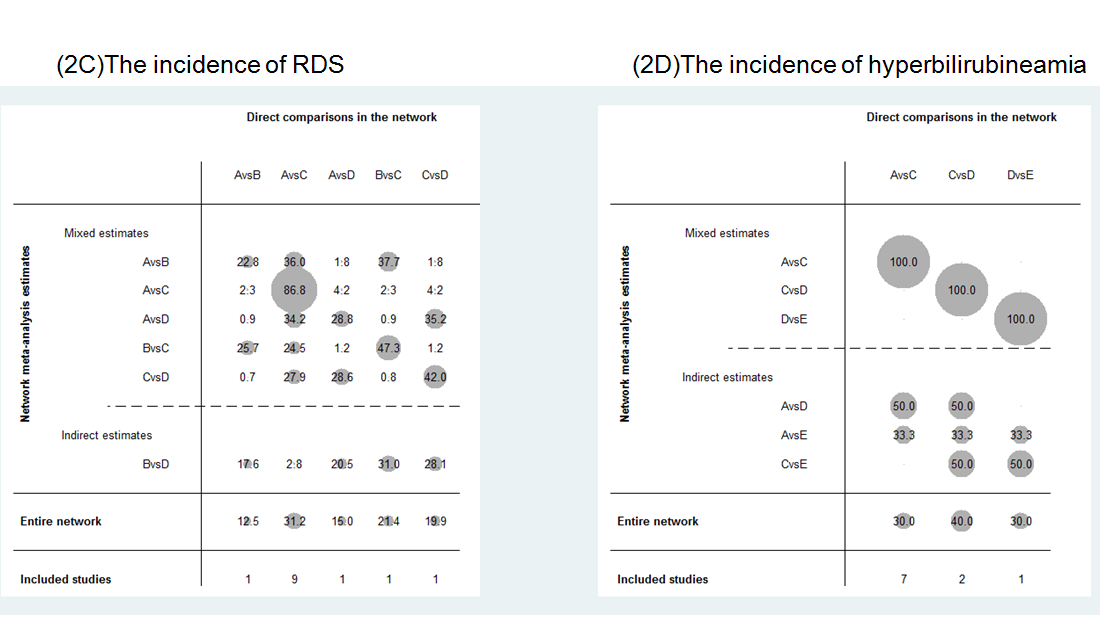


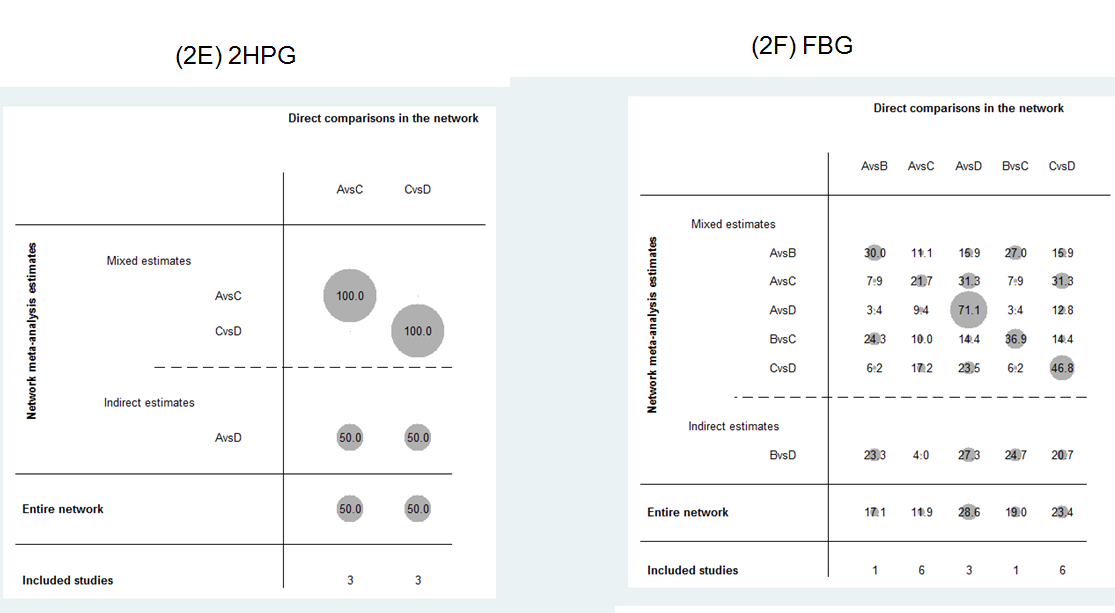


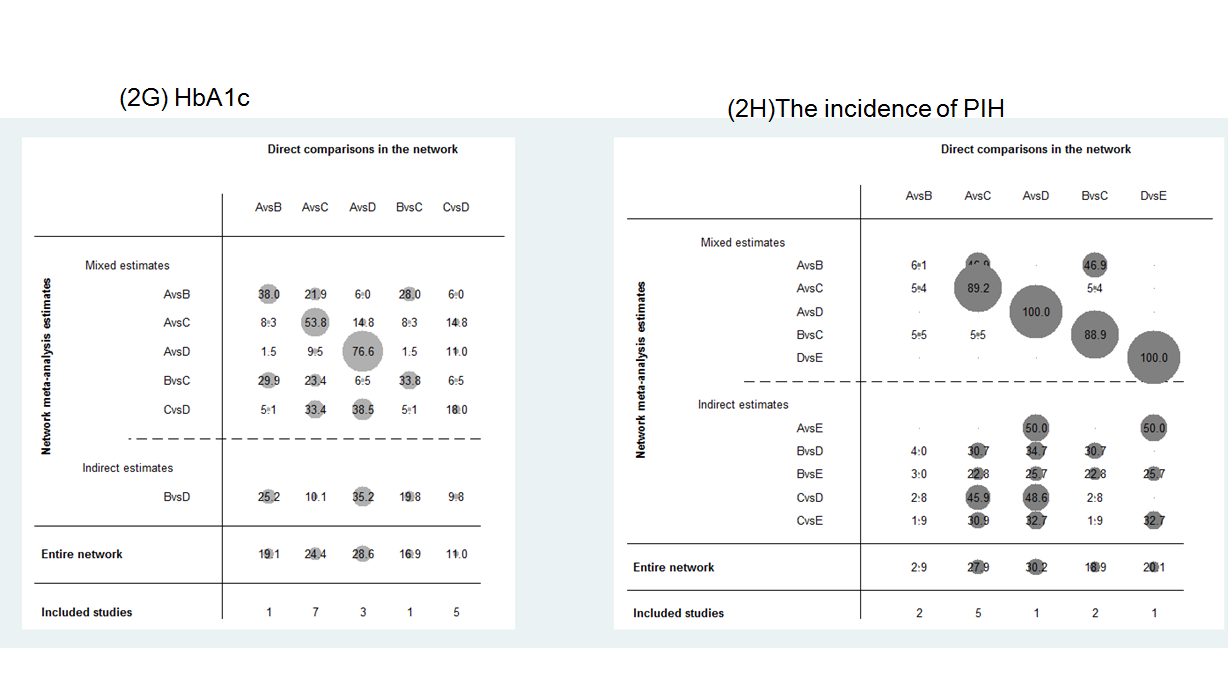


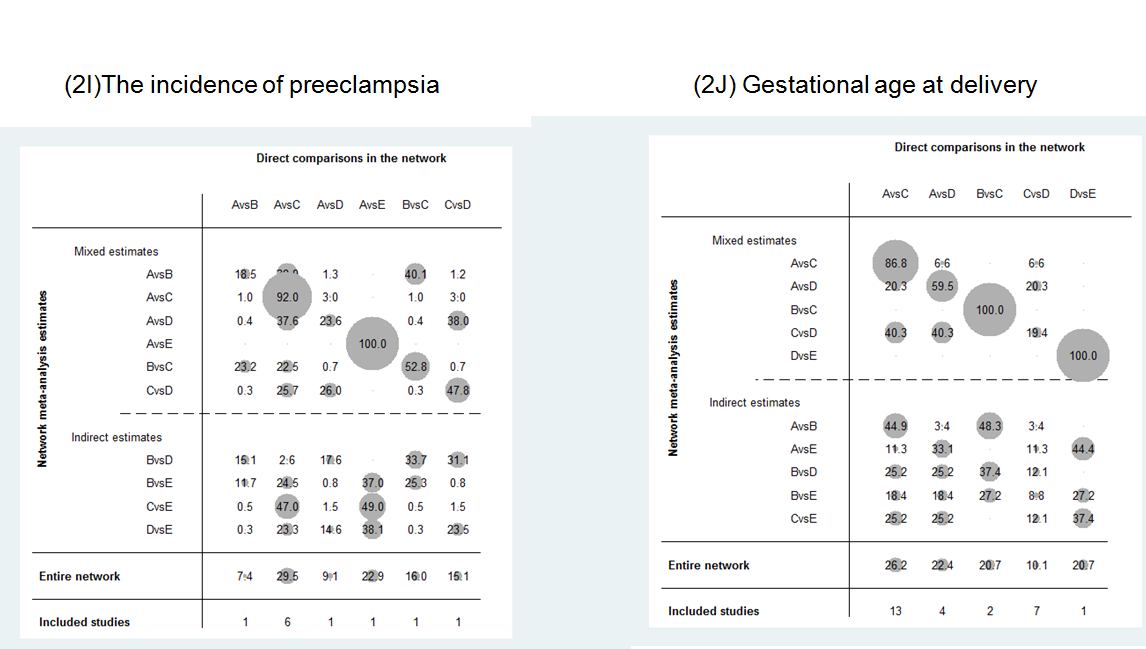


Appendix Figure 3:The cumulative probability plot of different treatments based on SUCRA values.


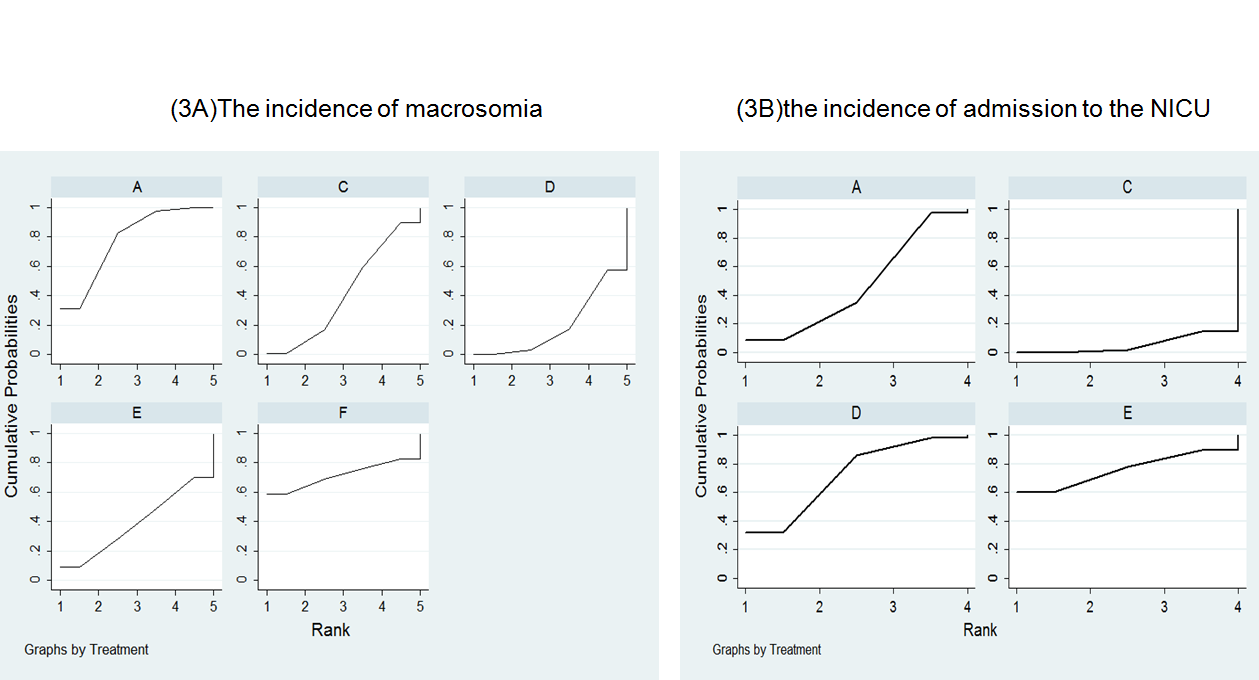


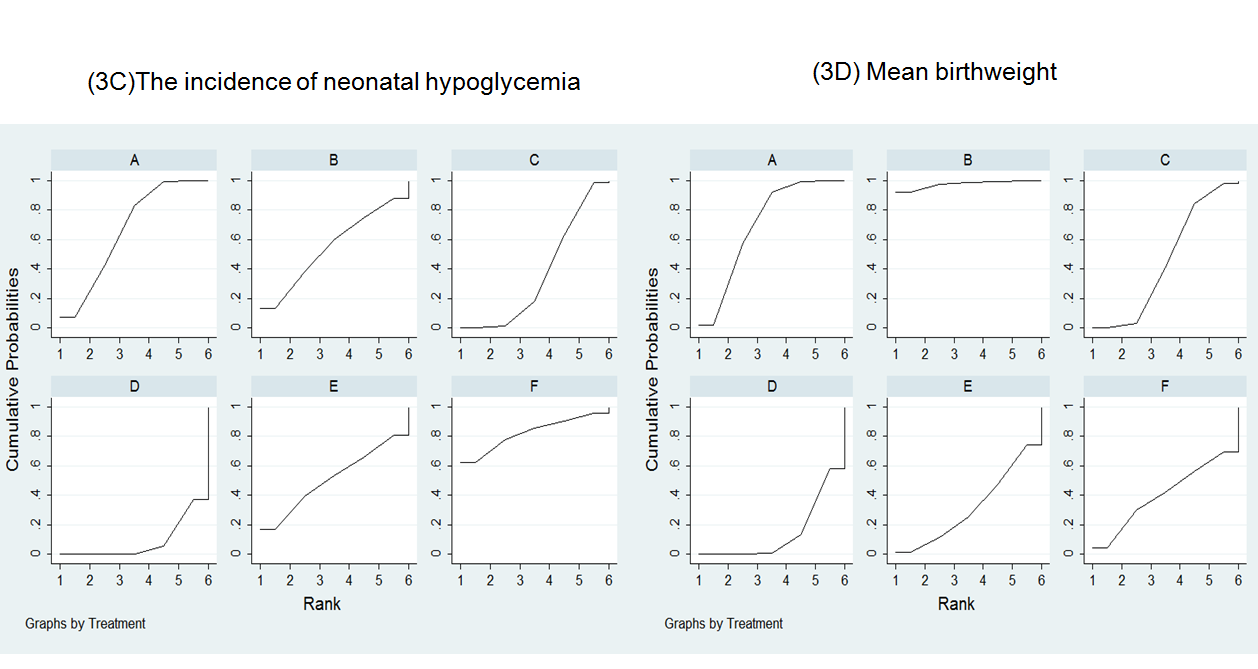


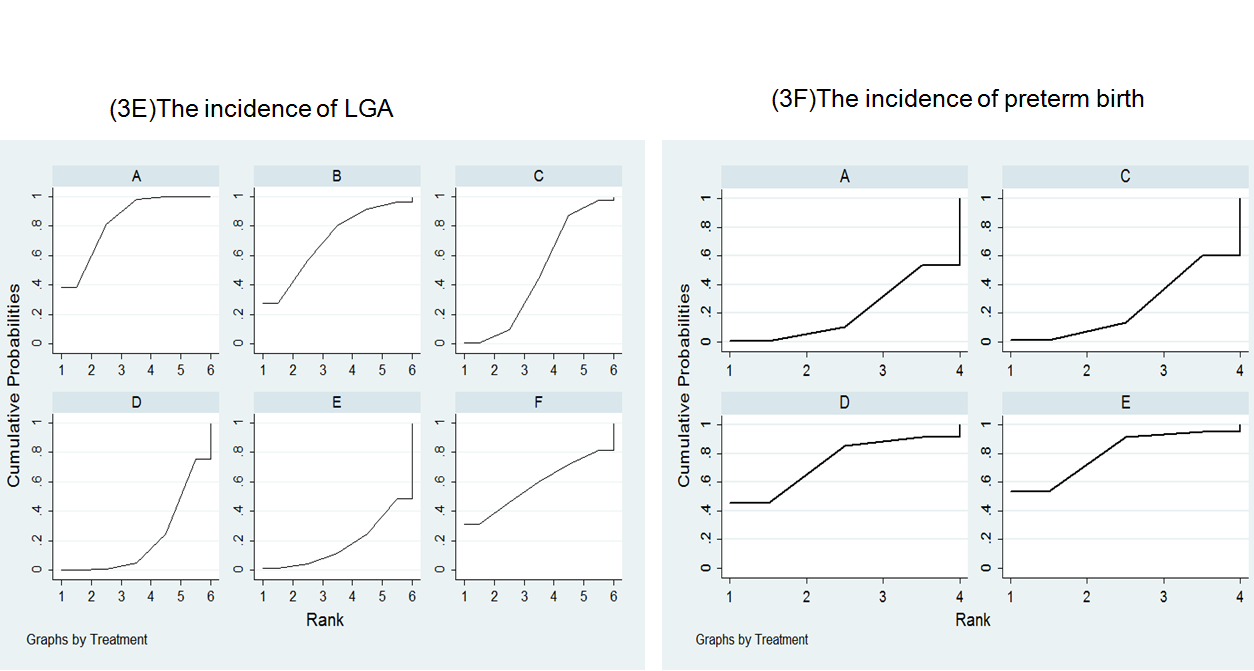


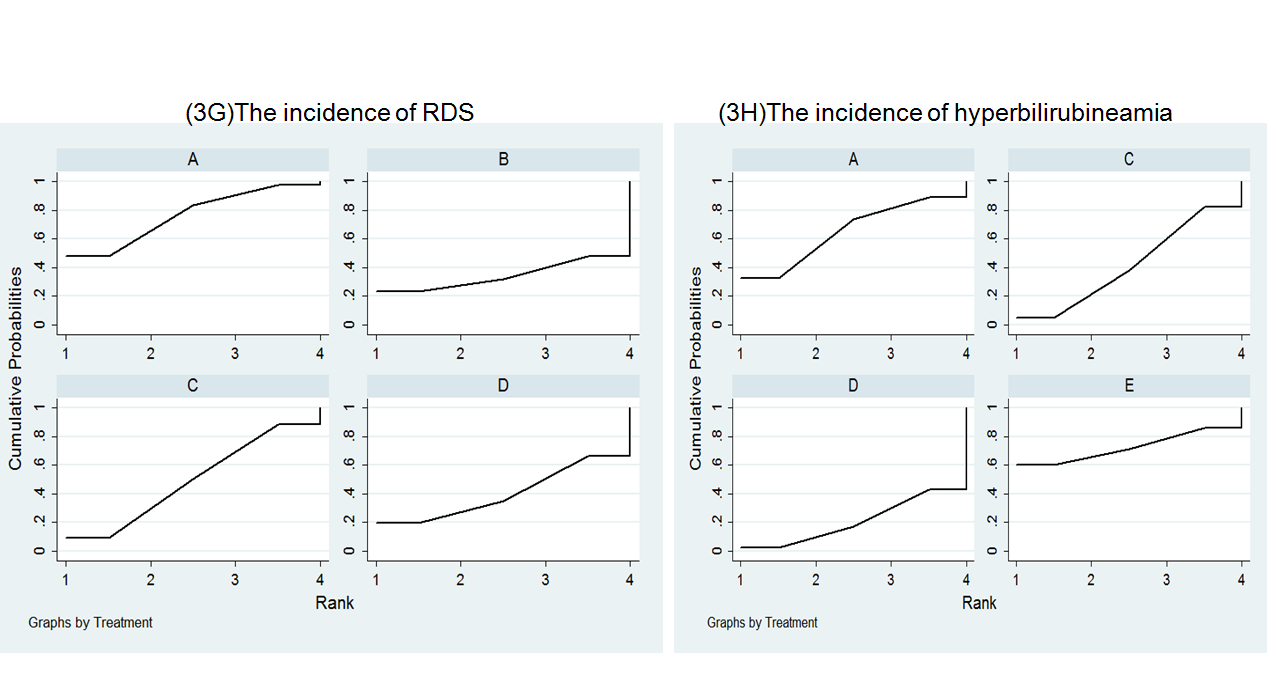


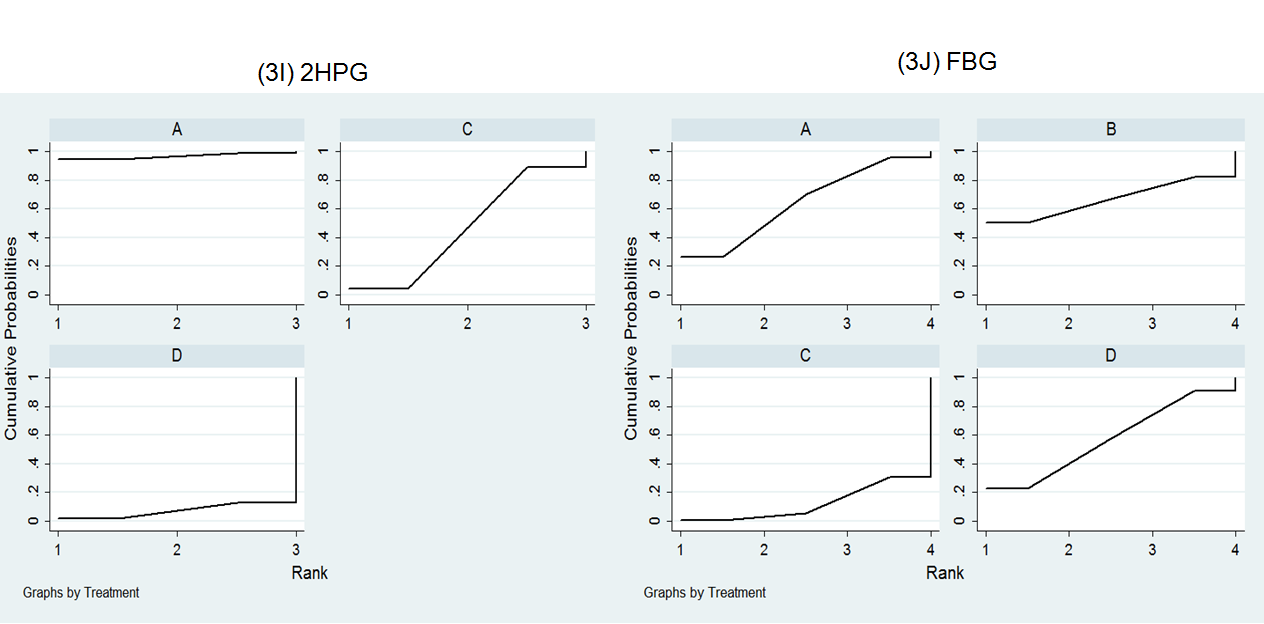


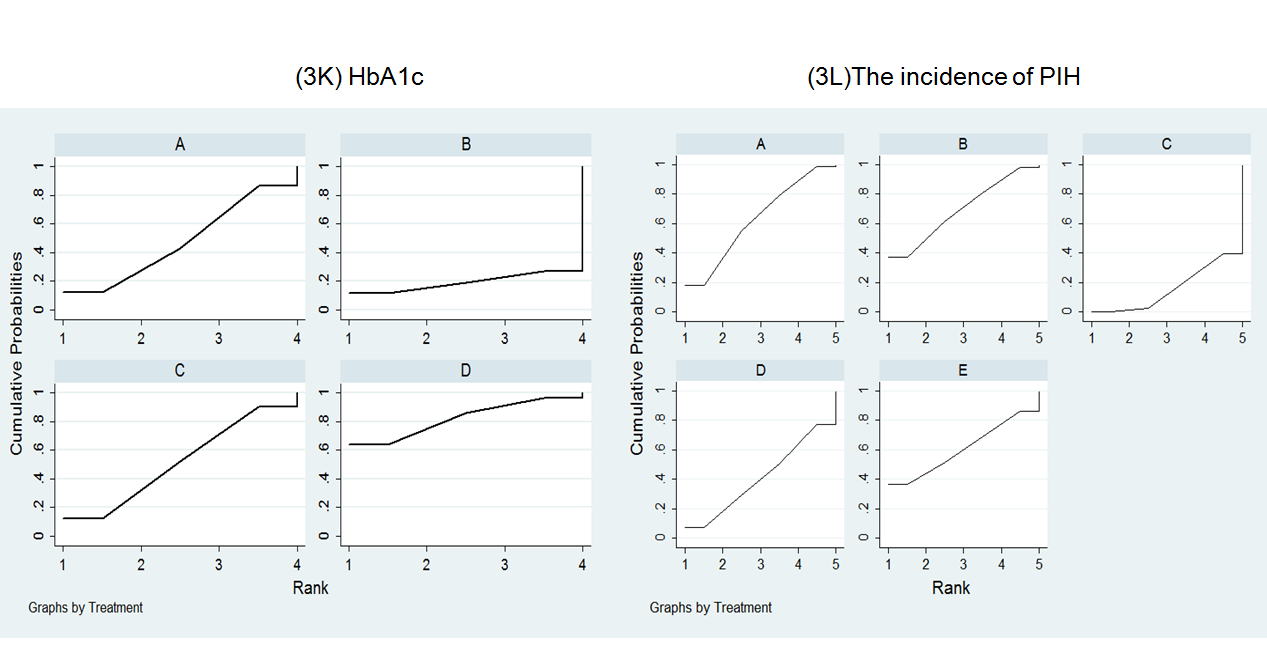


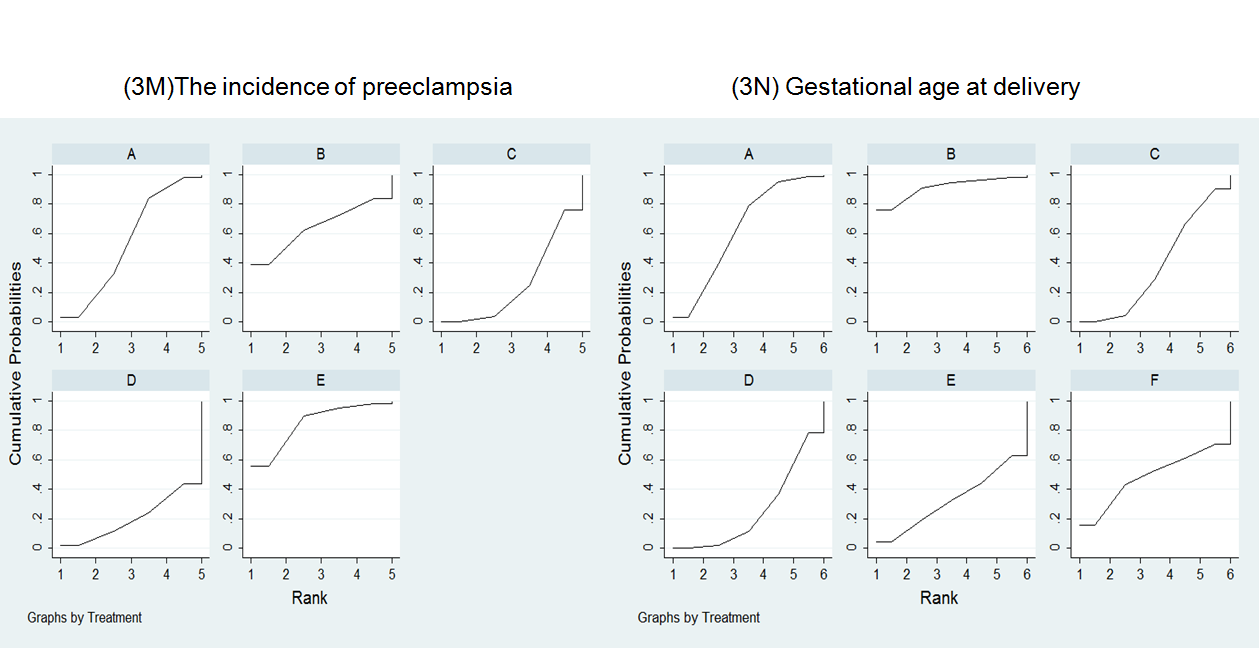


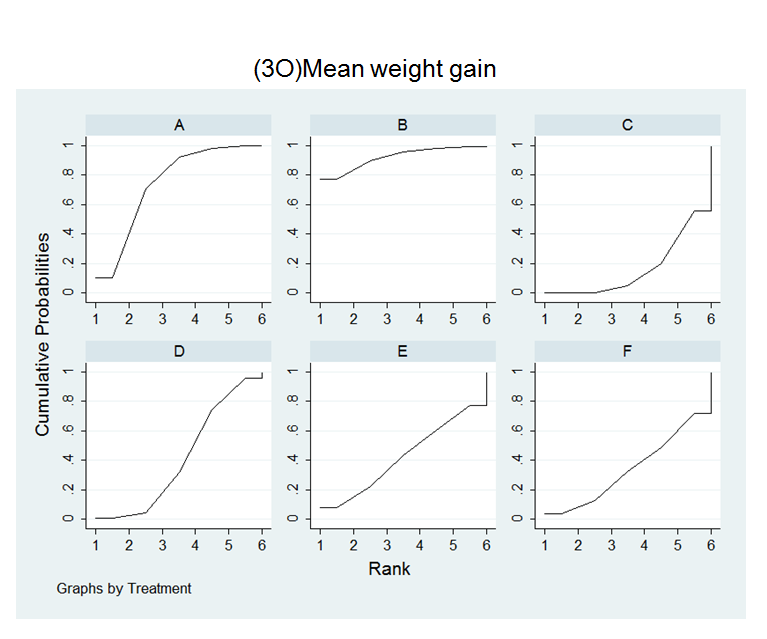


Appendix Figure 4:The comparision-ajusted funnel plot for each outcome from network meta-analysis.


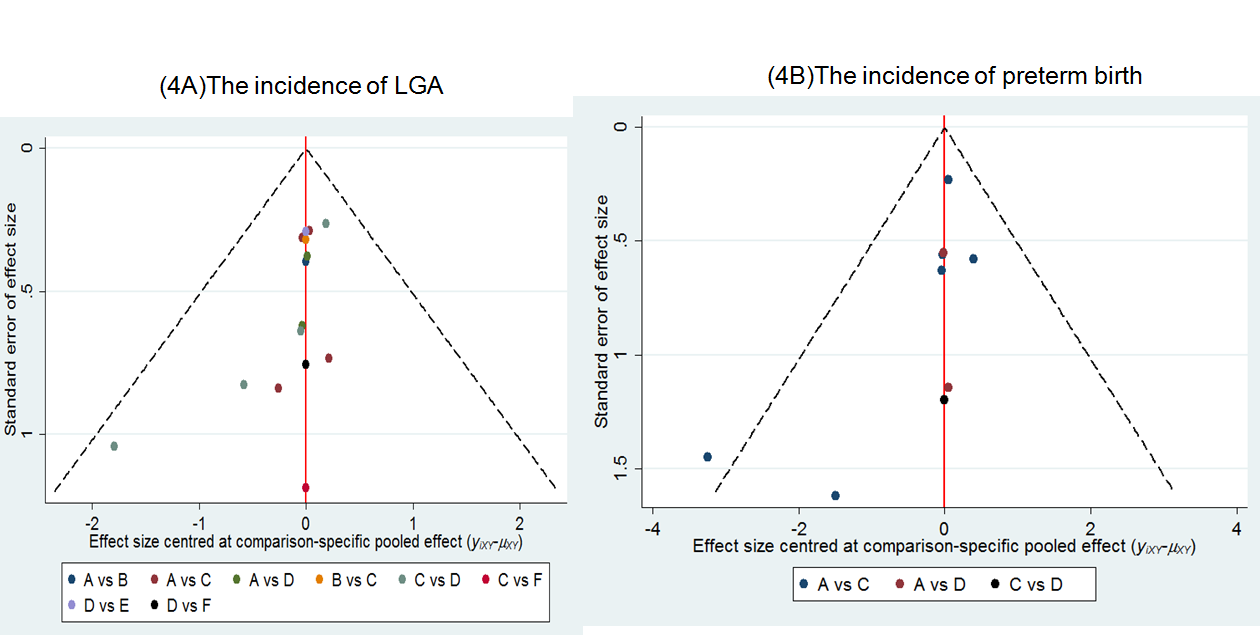


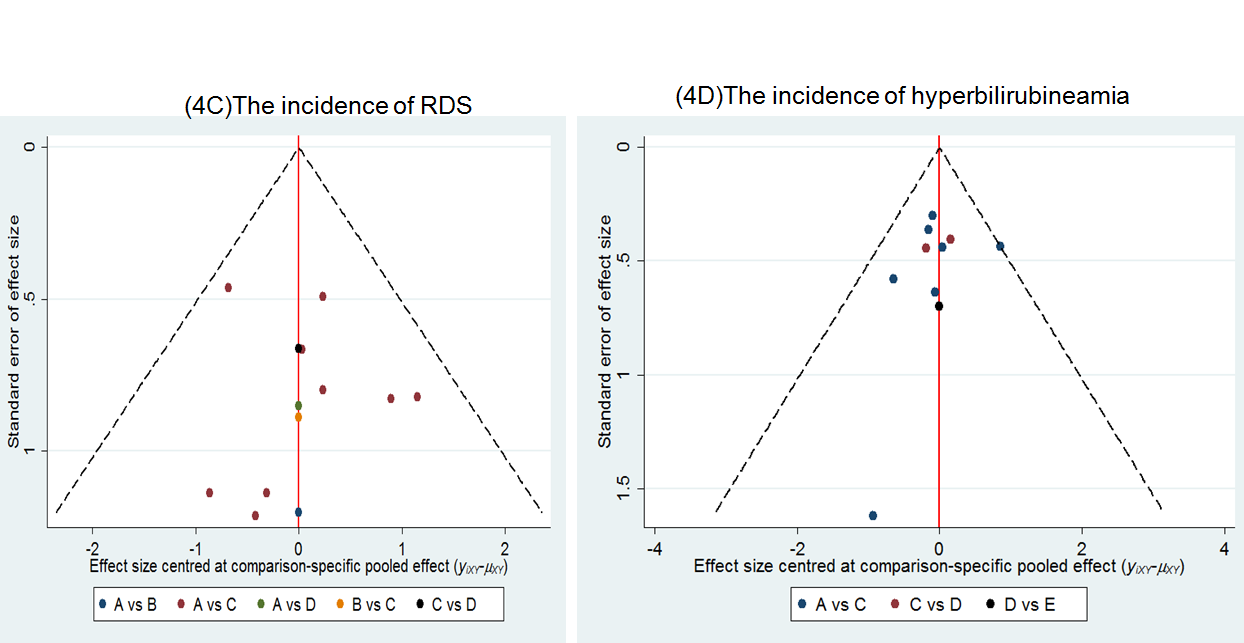


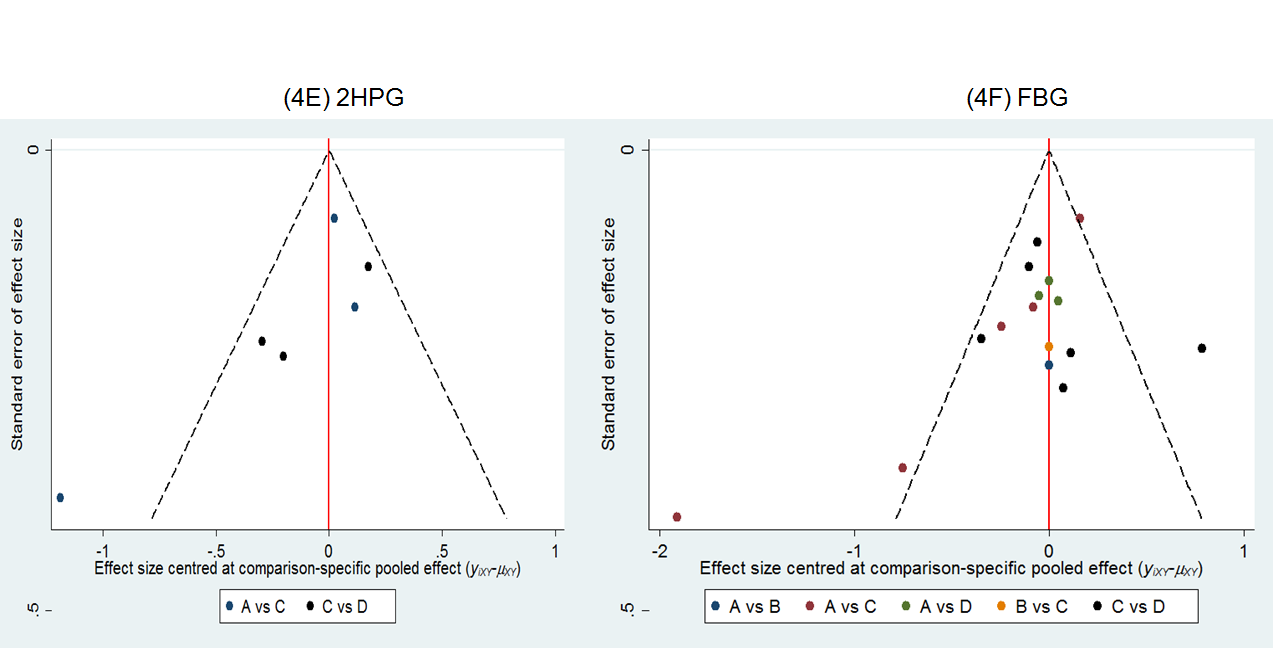


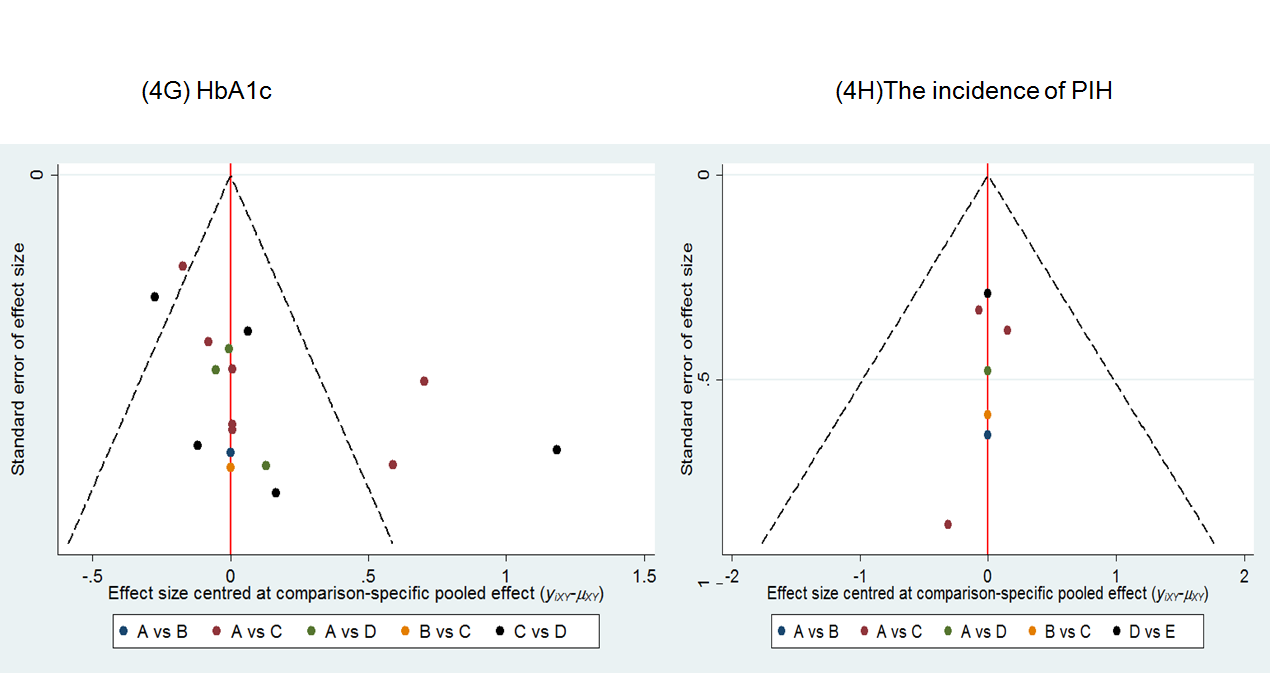


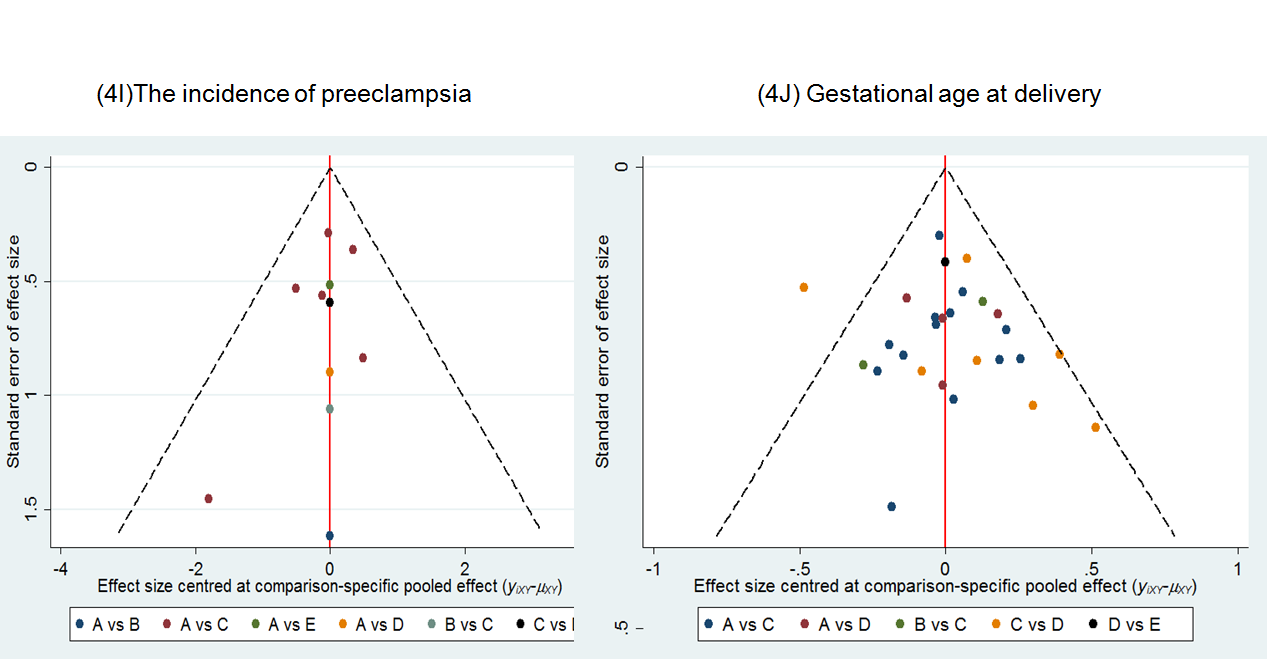


Appendix Figure 5:The inconsistency plot between direct and indirect comparisons for network meta-analysis.


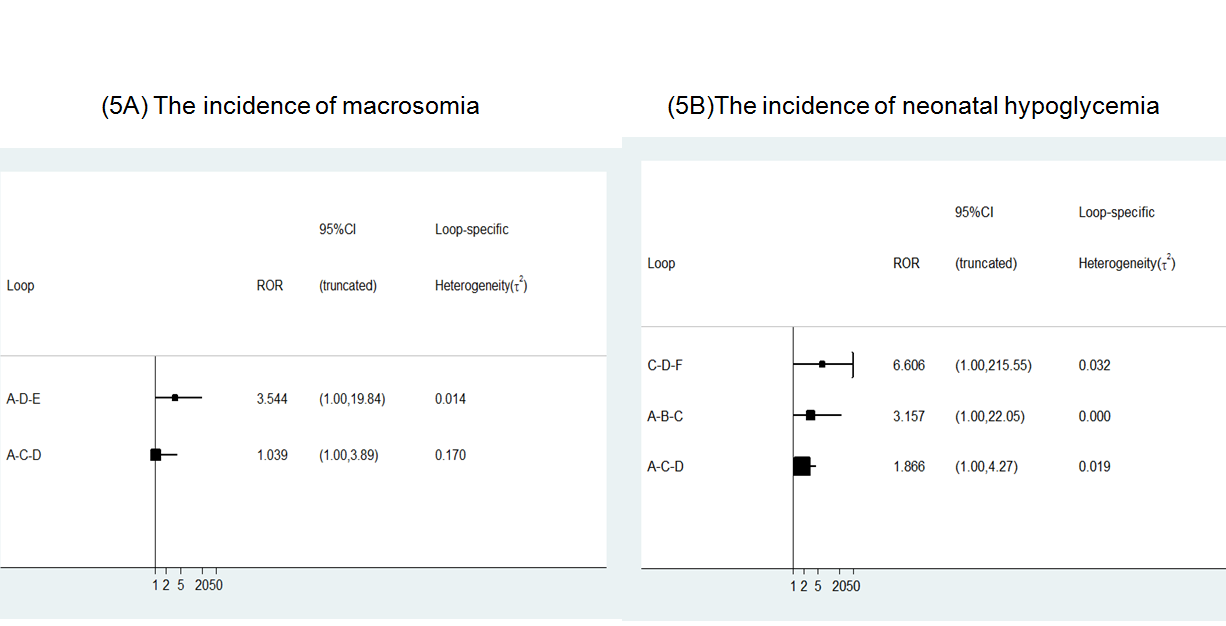


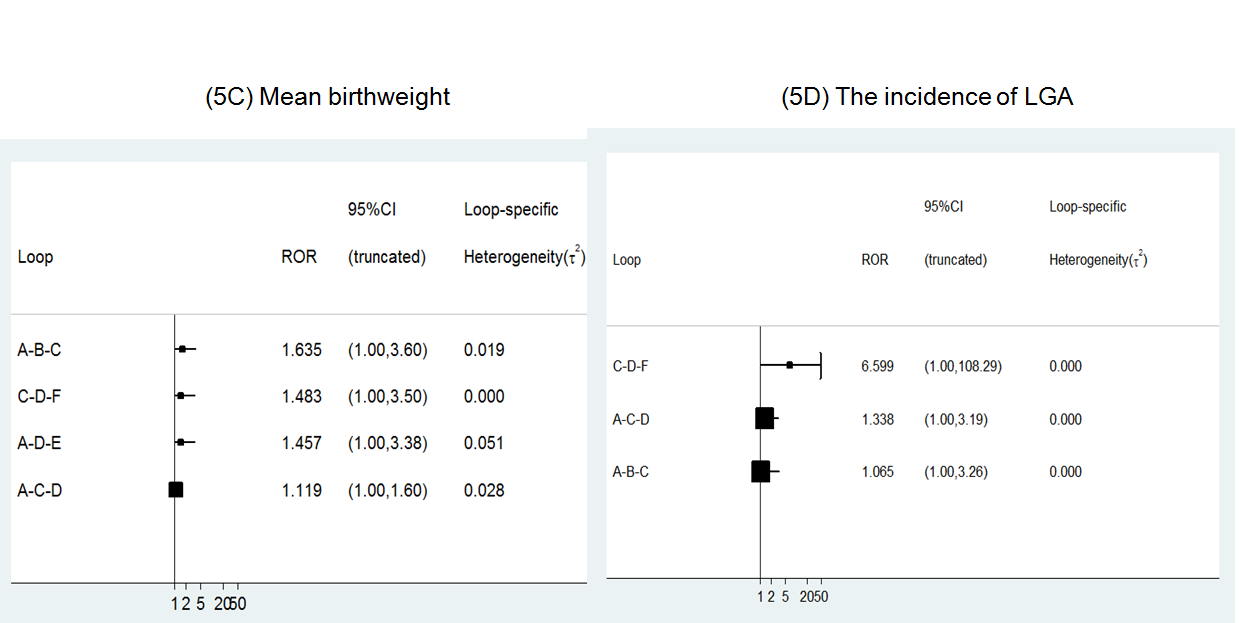


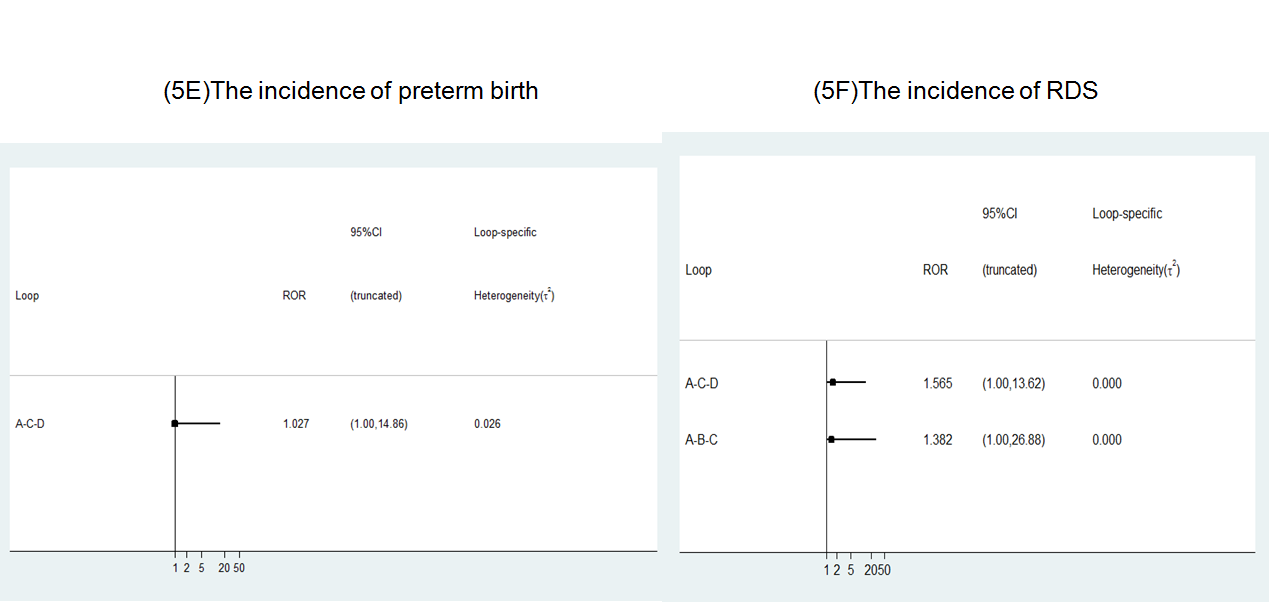


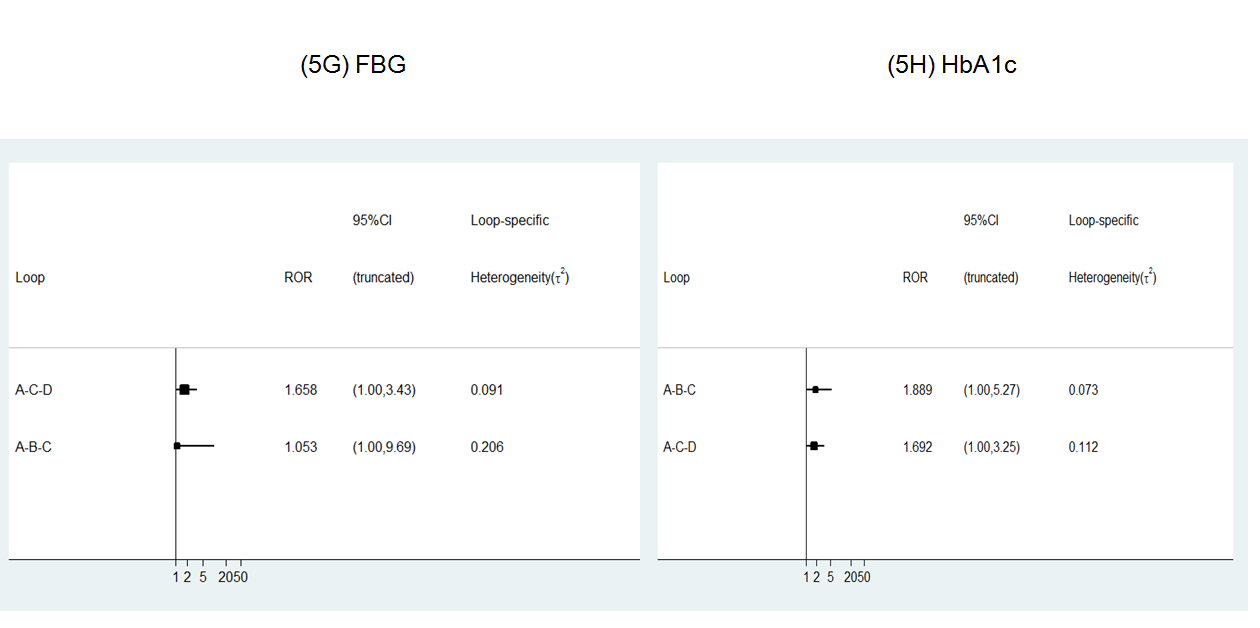


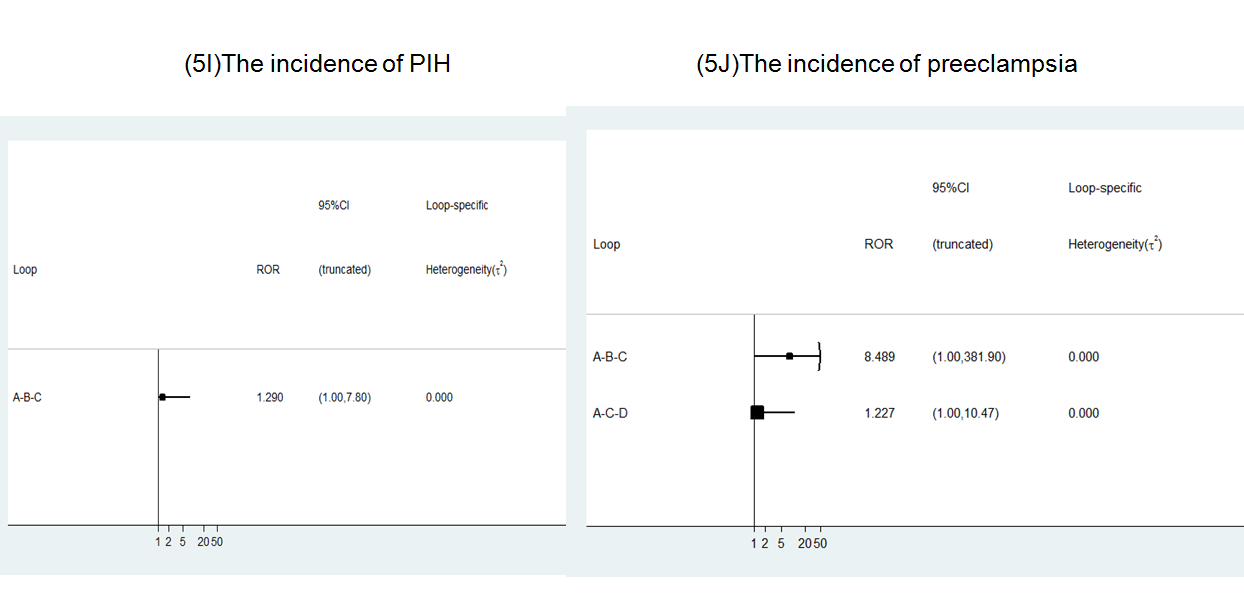


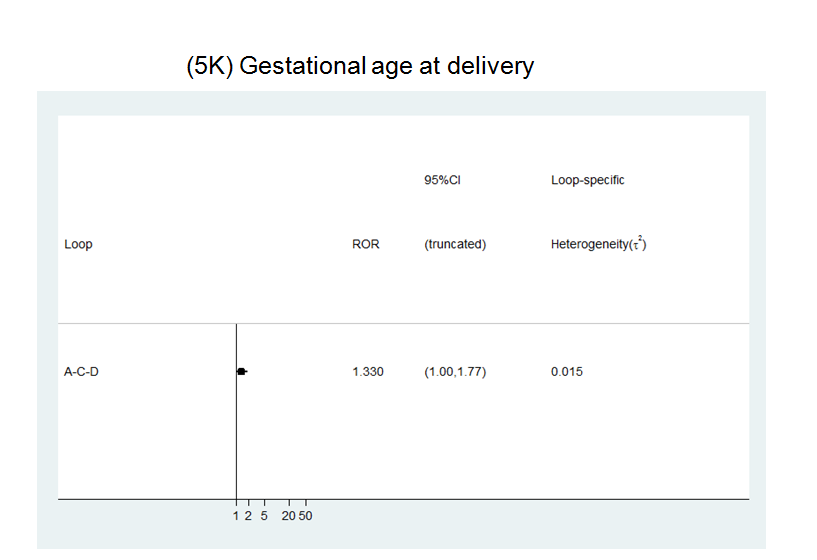


Appendix Table 1 :Results of Pairwise meta-analysis from pregnancy outcomes.

| Comparision group | number | RR(95%CI) | P | I^2^ | Egger’s Test, P |
| --- | --- | --- | --- | --- | --- |
| Pregnancy hypertention | | | | | |
| A vs B | 1 | 1.984(0.571-6.896) | 0.281 | - | - |
| A vs C | 3 | 0.658(0.411-1.052) | 0.080 | 0.0% | 0.705 |
| B vs C | 1 | 0.391（0.124-1.234） | 0.109 | - | - |
| D vs E | 1 | 1.243(0.705-2.191) | 0.452 | - | - |
| A vs D | 1 | 0.788(0.308-2.011) | 0.618 | - | - |
| preeclampsia | | | | | |
| A vs E | 1 | 2.071(0.755-5.680) | 0.157 | - | - |
| A vs B | 1 | 0.250(0.011-5.944) | 0.391 | - | - |
| A vs C | 6 | 0.761(0.529,1.095) | 0.142 | 0.0% | 0.679 |
| B vsC | 1 | 0.391(0.049-3.115) | 0.375 | - | - |
| A vs D | 1 | 0.658(0.113-3.824) | 0.641 | - | - |
| C vs D | 1 | 0.667(0.208-2.140) | 0.496 | - | - |
| macrosomia | | | | | |
| A vs E | 1 | 1.554(0.463-5.213) | 0.476 | - | - |
| C vs D | 8 | 0.788(0.510-1.219) | 0.285 | 60.8% | 0.553 |
| **A vs C** | **11** | **0.729(0.545,0.974)** | **0.033** | 0.0% | 0.092 |
| A vs D | 3 | 0.587(0.239-1.442) | 0.245 | 2.5% | 0.575 |
| D vs E | 1 | 0.711(0.359-1.409) | 0.328 | - | - |
| D vs F | 1 | 7.200(0.412-125.970) | 0.176 | - | - |
| LGA | | | | | |
| A vs B | 1 | 0.827(0.381-1.796) | 0.631 | - | - |
| A vs C | 4 | **0.647(0.438-0.956)** | **0.029** | 0.0% | 0.997 |
| B vs C | 1 | 0.753(0.403-1.410) | 0.376 | - | - |
| D vs E | 1 | 0.895(0.506-1.583) | 0.702 | - | - |
| C vs D | 4 | 0.838(0.542-1.295) | 0.426 | 25.4% | 0.034 |
| A vs D | 2 | **0.431(0.229-0.814)** | **0.009** | 0.0% | - |
| C vs F | 1 | 0.352(0.034-3.608) | 0.379 | - | - |
| D vs F | 1 | 2.375(0.539-10.463) | 0.253 | - | - |
| GA at delivery | | | | | |
| B vs C | 2 | **-0.284(-0.521,-0.048)** | **0.018** | 59.7% | - |
| A vs C | 13 | **-0.126(-0.212,-0.040)** | **0.004** | 0.0% | 0.997 |
| C vs D | 7 | **-0.180(-0.303,-0.057)** | **0.004** | 74.1% | 0.315 |
| A vs D | 4 | 0.009(-0.154,0.172) | 0.915 | 0.0% | 0.804 |
| D vs E | 1 | 0.000(-0.202,0.202) | 1.000 | - | - |
| Birth weight | | | | | |
| A vs E | 1 | 0.081(-0.362,0.524) | 0.719 | - | - |
| A vs C | 14 | **-0.111(-0.194,-0.028)** | **0.009** | 53.9% | 0.939 |
| A vs B | 1 | 0.225(-0.234,0.684) | 0.338 | - | - |
| B vs C | 1 | -0.800(-1.228,-0.372) | 0.000 | - | - |
| C vs D | 6 | **-0.180(-0.327,-0.033)** | **0.016** | 0.0% | 0.231 |
| A vs D | 4 | **-0.235(-0.399,-0.071)** | **0.005** | 63.8% | 0.897 |
| D vs E | 1 | -0.066(-0.268,0.137) | 0.524 | - | - |
| C vs F | 1 | -0.226(-0.815,0.363) | 0.452 | - | - |
| D vs F | 1 | 0.323(-0.283,0.929) | 0.296 | - | - |
| Weight gain | | | | | |
| A vs C | 5 | **-0.774(-0.928,-0.620)** | **0.000** | 94.9% | 0.037 |
| A vs B | 1 | 0.000(-0.458,0.458) | 1.000 | - | - |
| B vs C | 1 | **-2.257(-2.771,-1.743)** | **0.000** | - | - |
| D vs E | 1 | 0.000(-0.202,0.202) | 1.000 | - | - |
| C vs D | 2 | 0.037(-0.147,0.221) | 0.696 | 18.8% | - |
| A vs D | 2 | **-0.321(-0.560,-0.081)** | **0.009** | 0.0% | - |
| C vs F | 1 | 0.252(-0.337,0.842) | 0.401 | - | - |
| D vs F | 1 | -0.135(-0.738,0.467) | 0.660 | - | - |
| 2HPG | | | | | |
| A vs C | 3 | **-0.285(-0.417,-0.154)** | **0.000** | 80.8% | 0.480 |
| C vs D | 3 | **-0.302(-0.493,-0.111)** | **0.002** | 57.7% | 0.167 |
| FBG | | | | | |
| A vs B | 1 | 0.184(-0.274,0.643) | 0.430 | - | - |
| A vs C | 6 | -0.008(-0.122,0.105) | 0.888 | 85.4% | 0.002 |
| B vs C | 1 | **-0.523(-0.943,-0.103)** | **0.015** | - | - |
| C vs D | 6 | -0.080(-0.206,0.046) | 0.212 | 71.3% | 0.454 |
| A vs D | 3 | **0.192(0.018,0.366)** | **0.031** | 0.0% | 0.896 |
| HbA1C | | | | | |
| A vs C | 7 | -0.007(-0.108,0.093) | 0.884 | 79.6% | 0.099 |
| C vs D | 5 | 0.119(-0.013,0.252) | 0.078 | 89.0% | 0.245 |
| A vs B | 1 | 0.000(-0.444,0.444) | 1.000 | - | - |
| B vs C | 1 | 0.695(0.228,1.162) | 0.004 | - | - |
| A vs D | 3 | -0.130(-0.319,0.060) | 0.179 | 0.0% | 0.370 |
| preterm | | | | | |
| A vs D | 2 | **2.887(1.087,7.666)** | **0.033** | 0.0% | - |
| A vs C | 6 | 1.332(0.939,1.890) | 0.108 | 27.1% | 0.340 |
| C vs D | 1 | 2.000(0.191,20.969) | 0.563 | - | - |
| NICU admission | | | | | |
| AC | 11 | **0.772(0.644,0.927)** | **0.006** | 0.0% | 0.939 |
| AD | 3 | 1.805(0.863,3.778) | 0.117 | 0.0% | 0.118 |
| CD | 3 | 1.280(0.738,2.220) | 0.379 | 0.0% | 0.630 |
| DE | 1 | 1.163(0.535,2.530) | 0.703 | - | - |
| Neonatal hypoglycimia | | | | | |
| A vs C | 11 | **0.636(0.486,0.832)** | **0.001** | 0.0% | 0.704 |
| A vs D | 4 | 0.626(0.366,1.073) | 0.089 | 46.0% | 0.602 |
| C vs D | 6 | **0.647(0.423,0.991)** | **0.045** | 34.1% | 0.506 |
| D vs E | 1 | 1.968(0.365,10.617) | 0.431 | - | - |
| D vs F | 1 | 6.333(0.866,46.324) | 0.069 | - | - |
| A vs B | 1 | 0.372(0.073,1.90) | 0.236 | - | - |
| B vs C | 1 | 0.586(0.212,1.616) | 0.302 | - | - |
| C vs F | 1 | 0.704(0.047,10.565) | 0.799 | - | - |
| RDS | | | | | |
| A vs C | 9 | 0.785(0.503,1.224) | 0.285 | 0.0% | 0.569 |
| A vs B | 1 | 0.372(0.035,3.927) | 0.411 | - | - |
| B vs C | 1 | 1.563(0.274,8.908) | 0.615 | - | - |
| A vs D | 1 | 0.506(0.095,2.686) | 0.424 | - | - |
| C vs D | 1 | 1.000(0.274,3.656) | 1.000 | - | - |
| hyperbilirubineamia | | | | | |
| A vs C | 7 | 0.863(0.621,1.197) | 0.377 | 0.0% | 0.877 |
| C vs D | 2 | 0.791(0.440,1.424) | 0.435 | 0.0% | - |
| D vs E | 1 | 1.968(0.500,7.754) | 0.333 | - | - |

Notes: A=metformin, B=metformin plus insulin, C=insulin, D=glyburide, E=placebo, F=acarbose.

Appendix Table 2: ORs or SMD and 95%CI of 6 treatments according to the network meta-analysis.

| Preterm | | | | | | | | | | | | | | | | | | | | | | | | | | | | | | | | | | | | | |
| --- | --- | --- | --- | --- | --- | --- | --- | --- | --- | --- | --- | --- | --- | --- | --- | --- | --- | --- | --- | --- | --- | --- | --- | --- | --- | --- | --- | --- | --- | --- | --- | --- | --- | --- | --- | --- | --- |
| Ａ | | | | |  | | | | | |  | | | | |  | | | | | | | | |  | | | | | | | |  | | | | |
| 1.076(0.4501, 1.957) | | | | | **Ｃ** | | | | | |  | | | | |  | | | | | | | | |  | | | | | | | |  | | | | |
| 6.057(0.4624, 26.67) | | | | | 6.267(0.4844, 31.33) | | | | | | **Ｄ** | | | | |  | | | | | | | | |  | | | | | | | |  | | | | |
| 5.709(0.623, 24.2) | | | | | 6.247(0.583, 28.78) | | | | | | 2.837(0.07556, 16.19) | | | | | **Ｅ** | | | | | | | | |  | | | | | | | |  | | | | |
| RDS | | | | | | | | | | | | | | | | | | | | | | | | | | | | | | | | | | | | | |
| **Ａ** | | | | | | |  | | | | | | |  | | | | | | | | |  | | | | | | |  | | |  | | | | |
| 1.085（0.07228，6.146） | | | | | | | **Ｂ** | | | | | | |  | | | | | | | | |  | | | | | | |  | | |  | | | | |
| 0.8158（0.457，1.388） | | | | | | | 2.446（0.1473，10.44） | | | | | | | **Ｃ** | | | | | | | | |  | | | | | | |  | | |  | | | | |
| 0.8198（0.1597，2.558） | | | | | | | 2.612（0.09404，14.47） | | | | | | | 1.031（0.2182，3.047） | | | | | | | | | **Ｄ** | | | | | | |  | | |  | | | | |
| Hyperbilirubineamia | | | | | | | | | | | | | | | | | | | | | | | | | | | | | | | | | | | | | |
| **A** | | | | | |  | | | | | | | |  | | | | | | | |  | | | | | | |  | | | |  | | | | |
| 0.844(0.4534, 1.355) | | | | | | **C** | | | | | | | |  | | | | | | | |  | | | | | | |  | | | |  | | | | |
| 0.7285(0.2043, 1.737) | | | | | | 0.8556(0.3115, 1.83) | | | | | | | | **D** | | | | | | | |  | | | | | | |  | | | |  | | | | |
| 2.507(0.192, 13.35) | | | | | | 3.365(0.4497, 15.1) | | | | | | | | 2.964(0.2624, 15.41) | | | | | | | | **E** | | | | | | |  | | | |  | | | | |
| Pregnancy hypertention | | | | | | | | | | | | | | | | | | | | | | | | | | | | | | | | | | | | | |
| **Ａ** |  | | | | | | | | | | | | |  | | | | |  | | | | | | | | |  | | | | | | |  | | |
| 1.23(0.4531, 2.824) | **Ｂ** | | | | | | | | | | | | |  | | | | |  | | | | | | | | |  | | | | | | |  | | |
| 0.5819(0.2963, 1.052) | 0.5423(0.216, 1.081) | | | | | | | | | | | | | **Ｃ** | | | | |  | | | | | | | | |  | | | | | | |  | | |
| 0.9736(0.1921, 2.912) | 0.9684(0.1302, 3.453) | | | | | | | | | | | | | 1.852(0.3094, 5.987) | | | | | **Ｄ** | | | | | | | | |  | | | | | | |  | | |
| 1.456(0.1666, 5.352) | 1.462(0.1156, 5.972) | | | | | | | | | | | | | 2.806(0.2767, 11.37) | | | | | 1.491(0.4351, 3.787) | | | | | | | | | **Ｅ** | | | | | | |  | | |
| Preeclampsia | | | | | | | | | | | | | | | | | | | | | | | | | | | | | | | | | | | | | |
| **Ａ** | | | |  | | | | | | | | |  | | | | | | | |  | | | | | | | | | | |  | | | |  | |
| 6.648（0.1853，39.86） | | | | **Ｂ** | | | | | | | | |  | | | | | | | |  | | | | | | | | | | |  | | | |  | |
| 0.7416（0.4063，1.193） | | | | 0.7977（0.01711，3.605） | | | | | | | | | **Ｃ** | | | | | | | |  | | | | | | | | | | |  | | | |  | |
| 0.6792（0.129，2.074） | | | | 0.7478（0.008815，3.671） | | | | | | | | | 0.9349（0.1879，2.614） | | | | | | | | **Ｄ** | | | | | | | | | | |  | | | |  | |
| 3.134（0.5886，10.46） | | | | 3.735（0.04315，20.76） | | | | | | | | | 4.583（0.7571，15.62） | | | | | | | | 8.005（0.7143，34.9） | | | | | | | | | | | **Ｅ** | | | |  | |
| Gestational age at delivery | | | | | | | | | | | | | | | | | | | | | | | | | | | | | | | | | | | | | |
| **A** | |  | | | | | | | |  | | | | | | | |  | | | | | | | | | | | | |  | | | | | |  |
| 0.3388(-0.1601, 0.8278) | | **B** | | | | | | | |  | | | | | | | |  | | | | | | | | | | | | |  | | | | | |  |
| -0.07685(-0.204,0.04704) | | -0.4156(-0.9069, 0.07787) | | | | | | | | **C** | | | | | | | |  | | | | | | | | | | | | |  | | | | | |  |
| -0.1218(-0.285, 0.05562) | | -0.4606(-0.9608, 0.05736) | | | | | | | | -0.04493(-0.2023, 0.1189) | | | | | | | | **D** | | | | | | | | | | | | |  | | | | | |  |
| -0.1222(-0.5478 ,0.316) | | -0.4609(-0.4609, 0.2061) | | | | | | | | -0.0453(-0.4714,0.3898) | | | | | | | | -3.722E-4(-0.395, 0.3971) | | | | | | | | | | | | | **E** | | | | | |  |
| -0.02699(-0.6635, 0.6028) | | -0.3658(-1.131, 0.4263) | | | | | | | | 0.04986(-0.5773,0.6685) | | | | | | | | 0.09479(-0.5323, 0.7241) | | | | | | | | | | | | | 0.09516(-0.622, 0.8371) | | | | | | **F** |
| 2HPG | | | | | | | | | | | | | | | | | | | | | | | | | | | | | | | | | | | | | |
| **A** | |  | | | | | |  | | | | | | |  | | | | | | | | |  | | | | | | | | | |  | | | |
| -0.5939(-1.337, 0.1288) | | **C** | | | | | |  | | | | | | |  | | | | | | | | |  | | | | | | | | | |  | | | |
| -0.9876(-2.022, 0.001284) | | -0.3937(-1.13,0.3407) | | | | | | **D** | | | | | | |  | | | | | | | | |  | | | | | | | | | |  | | | |
| FBG | | | | | | | | | | | | | | | | | | | | | | | | | | | | | | | | | | | | | |
| **A** | | |  | | | | | | | | |  | | | | | | | |  | | | | | | |  | | | | | | |  | | | |
| 0.05797(-0.5853, 0.6988) | | | **B** | | | | | | | | |  | | | | | | | |  | | | | | | |  | | | | | | |  | | | |
| -0.2173(-0.5207, 0.07124) | | | -0.2753(-0.9264, 0.3434) | | | | | | | | | **C** | | | | | | | |  | | | | | | |  | | | | | | |  | | | |
| -0.0304(-0.4079, 0.3238) | | | -0.08837(-0.784, 0.5946) | | | | | | | | | 0.1869(-0.1249, 0.5067) | | | | | | | | D | | | | | | |  | | | | | | |  | | | |
| HbA1C | | | | | | | | | | | | | | | | | | | | | | | | | | | | | | | | | | | | | |
| A | |  | | | | | | |  | | | | | | | |  | | | | | | | | |  | | | | | | | |  | | | |
| -0.3092(-1.177, 0.5519) | | B | | | | | | |  | | | | | | | |  | | | | | | | | |  | | | | | | | |  | | | |
| 0.02208(-0.2923, 0.348) | | 0.3313(-0.519, 1.19) | | | | | | | C | | | | | | | |  | | | | | | | | |  | | | | | | | |  | | | |
| 0.168(-0.2236, 0.5638) | | 0.4773(-0.4246, 1.387) | | | | | | | 0.146(-0.2158, 0.5072) | | | | | | | | D | | | | | | | | |  | | | | | | | |  | | | |

Notes: A=metformin, B=metformin plus insulin, C=insulin, D=glyburide, E=placebo, F=acarbose.

Appendix Table 3: Results of NMA about the efficacy of OADs between GDM patients with and without obesity.

| 2HPG |  |  |
| --- | --- | --- |
| A |  |  |
| -0.5998(-1.38, 0.1512 ) | C |  |
| -1.141(-2.372, 0.0706 ) | -0.5413( -1.49, 0.4147) | D |
| FBG |  |  |
| A | 0.1185(-0.3471, 0.5768) | 0.08608(-0.3064, 0.4742) |
| -0.4235( -1.153, 0.2744) | C | -0.03242(-0.4317, 0.3787) |
| -0.3301(-1.355, 0.6626) | 0.09336( -0.8079, 0.9994) | D |
| HbA1c |  |  |
| A | 0.05061(-0.3522, 0.4592) | 0.03753(-0.3711,0.4527) |
| -0.2252(-1.028, 0.5842) | C | -0.01307(-0.4639, 0.4485) |
| 0.2861(-0.6501, 1.227) | 0.5113(-0.3088, 1.332) | D |

treatment the efficacy of OADs in obese GDM patients the efficacy of OADs in non-obese GDM patients Notes: A=metformin, C=insulin, D=glyburide, 2HPG=2-hour post-prandial glucose, FBG= Fasting blood glucose,

HbA1c =Glycohemoglobin, NA: not available.

Appendix Table 4: SUCRA values about the efficacy of OADs between GDM patients with and without obesity.

| Treatment | Obese GDM patients | | | Non-obese GDM patients | | |
| --- | --- | --- | --- | --- | --- | --- |
|  | 2HPG | FBG | HbA1c | 2HPG | FBG | HbA1c |
| A | 96.5 | 85.3 | 60.5 | NA | 33.2 | 51.0 |
| C | 49.1 | 29.5 | 19.9 | NA | 76.2 | 70.7 |
| D | 8.5 | 53.0 | 86.4 | NA | 66.8 | 68.3 |

Notes: A=metformin, C=insulin, D=glyburide, 2HPG=2-hour post-prandial glucose, FBG= Fasting blood glucose,

HbA1c =Glycohemoglobin, NA: not available.

**NMA model description**

1. Random Effects Model for Continuous Data in WinBUGS
y=a table of the arm-means, sd=a table of the arm sd, n=a table of the arm sample size, t=a table with
the names (numbers) of treatments, na=a vector with the number of arms in each study, ref=a number
specifying which is the reference treatment.

model{
for(i in 1:ns){
w[i,1] <- 0
delta[i,t[i,1]]<-0
u[i] ~ dnorm(0,.0001)
for (k in 1:na[i]) {
se[i,t[i,k]]<- sd[i,t[i,k]]/sqrt(n[i,t[i,k]])
var[i,t[i,k]]<- se[i,t[i,k]]*se[i,t[i,k]]
prec[i,t[i,k]]<- 1/var[i,t[i,k]]
#normal likelihood
y[i,t[i,k]] ~ dnorm(phi[i,t[i,k]],prec[i,t[i,k]])
phi[i,t[i,k]]<- (u[i]+delta[i,t[i,k]])*pooled.sd[i]
#calculate the pooled SD
nom1[i,k]<- n[i,t[i,k]]*sd[i,t[i,k]]*sd[i,t[i,k]] #nominator for the pooled sd
}
ss[i]<- sum(n[i,1:nt])-nt+na[i] #total sample size in a study
nom[i]<- sum(nom1[i,1:na[i]]) #nominator for the pooled sd
pooled.sd[i]<- sqrt(nom[i]/(ss[i]-na[i])) #pooled sd
for (k in 2:na[i]) {
delta[i,t[i,k]] ~ dnorm(md[i,t[i,k]],taud[i,t[i,k]]) # trial-specific SMD distributions
md[i,t[i,k]]<- d[t[i,k]]-d[t[i,1]]+sw[i,k] # mean of SMD distributions
taud[i,t[i,k]]<- tau*2*(k-1)/k #precision of SMD distributions
w[i,k] <- (delta[i,t[i,k]]-d[t[i,k]]+d[t[i,1]]) #adjustment, multi-arm RCTs
sw[i,k] <- sum(w[i,1:k-1])/(k-1) } # cumulative adjustment for multi-arm trials
}
d[ref]<-0
for (k in 2:nt) {d[k] ~ dnorm(0,.0001) }
SD~dunif(0,1) #vague prior for random effects standard deviation
tau<-1/pow(SD,2)
10
# Collection of results#
# pairwise SMDs
# for all comparisons
for (c in 1:(nt-1)) { for (k in (c+1):nt) { SMD[c,k] <- d[c] - d[k] } #to have negative values
}
#Fit of the Model#
for(i in 1:ns) {
for(k in 1:na[i]) {
Darm[i,k]<-(y[i,t[i,k]]-phi[i,t[i,k]])*(y[i,t[i,k]]-phi[i,t[i,k]])/var[i,t[i,k]]
}
D[i]<- sum(Darm[i,1:na[i]])
}
D.bar<- sum(D[])
}

2. Random Effects Model for Dichotomous Data in WinBUGS
r= a table of the number of events, n=a table of the arm sample size, t=a table with the names (numbers)
of treatments, na=a vector with the number of arms in each study, ref=a number specifying which is the
reference treatment

model {
for(i in 1:ns) {
w[i,1]<- 0
delta[i,t[i,1]]<- 0
#Binomial Likelihood#
for (k in 1:na[i]) {
r[i,t[i,k]] ~ dbin(p[i,t[i,k]],n[i,t[i,k]])
}
#Parameterization of the model#
logit(p[i,t[i,1]])<- mu[i]
for (k in 2:na[i]) {
logit(p[i,t[i,k]])<- mu[i] + delta[i,t[i,k]]
delta[i,t[i,k]] ~ dnorm(md[i,t[i,k]],taud[i,t[i,k]])
taud[i,t[i,k]]<- tau *2*(k-1)/k
md[i,t[i,k]]<-d[t[i,k]] - d[t[i,1]] + sw[i,k]
w[i,k]<- (delta[i,t[i,k]] - d[t[i,k]] + d[t[i,1]])
sw[i,k]<- sum(w[i,1:k-1])/(k-1)
} }
#Priors#
sd ~ dnorm(0,1)I(0,1)
11
tau<- 1/pow(sd,2)
for(k in 1:(ref-1)) {
d[k] ~ dnorm(0,.0001)
}
for(k in (ref+1):nt) {
d[k] ~ dnorm(0,.0001)
}
for(i in 1:ns) {
mu[i] ~ dnorm(0,.0001)
}
# Collection of results#
#Estimated & Predicted Odds Ratios#
d[ref]<- 0
for(i in 1:(nt-1)) {
for (j in (i+1):nt) {
OR[i,j]<- exp(d[i] - d[j])
LOR[i,j]<- d[i] - d[j]
} }
#Fit of the Model#
for(i in 1:ns) {
for (k in 1:na[i]) {
Darm[i,k]<- -2*( r[i,t[i,k]] *log(n[i,t[i,k]]*p[i,t[i,k]]/ r[i,t[i,k]])+(n[i,t[i,k]] -
r[i,t[i,k]])*log((n[i,t[i,k]]-n[i,t[i,k]]* p[i,t[i,k]])/(n[i,t[i,k]]- r[i,t[i,k]])))
}
D[i]<- sum(Darm[i,1:na[i]])
}
D.bar<- sum(D[])
}
